# Alpha1-antitrypsin protects lung cancer cells from staurosporine-induced apoptosis: the role of bacterial lipopolysaccharide

**DOI:** 10.1038/s41598-020-66825-w

**Published:** 2020-06-12

**Authors:** Natalie Schwarz, Srinu Tumpara, Sabine Wrenger, Evrim Ercetin, Jürg Hamacher, Tobias Welte, Sabina Janciauskiene

**Affiliations:** 1 Department of Internal Medicine, Biomedical Research in Endstage and Obstructive Lung Disease Hannover (BREATH), Member of the German Center for Lung Research (DZL), Hannover Medical School, 30625 Hannover, Germany; 2 Pneumology, Clinic for General Internal Medicine, Lindenhofspital Bern, 3012 Bern, Switzerland; 3 Lungen-und Atmungsstiftung, Bern, 3012 Bern, Switzerland

**Keywords:** Cancer, Oncology, Risk factors

## Abstract

Elevated levels of plasma alpha1-antitrypsin (AAT) correlate with a poor prognosis of various cancers. Herein, we investigated effects of exogenous AAT on non-small lung cancer cell lines with high (H1975) and very low (H661) baseline expression of *SERPINA1* gene encoding AAT protein. Comparison of cells grown for 3 weeks in a regular medium versus medium supplemented with 2 mg/ml of AAT revealed that in the presence of AAT cells acquire better proliferative properties, resistance to staurosporine (STS)-induced apoptosis, and show higher expression of *CLU*, a pro-tumorigenic gene coding clusterin protein. Similarly, the co-administration of STS with AAT or addition of AAT to the cells pre-treated with STS abrogated effects of STS in both cell lines. Following experiments with H1975 cells have shown that AAT blocks critical steps in STS-induced cell death: inhibition of AKT/MAPK pathways, and activation of caspase 3 and autophagy. AAT does not inhibit apoptosis-triggered by chloroquine (inhibitor of autophagy) or streptonigrin (inducer of p53 pathway). The anti-apoptotic effects of AAT were unaffected by lipopolysaccharide (LPS). However, AAT induced TLR4 levels and enhanced LPS effects on the production of IL-6, a tumor-promoting cytokine. Our data provide further evidence that AAT plays a significant role in the tumorigenesis.

## Introduction

In recent years, an importance of the inflammation in cancer progression became more recognised^1^. In both early and advanced stages of cancer, the development and maintenance of a systemic inflammation confer poorer outcome^2^. Repeated studies show that neutrophil, lymphocyte and platelet counts, and acute phase proteins, such as C-reactive protein and albumin and their combinations, have a prognostic value^3–5^. Besides, the major hallmark of human cancers is the resistance to apoptosis and treatments, since most current anticancer therapies, including chemotherapy, radio- and immunotherapy, primarily act by activating cell death pathways^6^. Different research groups reported that acute phase proteins interfere with apoptosis induction. For example, α1-acid glycoprotein expresses a direct anti-apoptotic mode on tumor necrosis factor (TNF)-α-induced apoptosis^7^. The C-reactive protein enhances secretion of interleukin 6 (IL-6) and, synergized with IL-6, protects cancer cells from chemotherapy-induced apoptosis^8,9^.

There are observations that certain members of serine protease inhibitor (serpin) superfamily, such as plasminogen activator inhibitor-1, are also associated with poor outcome in several types of cancer^10^. Alpha1-antitrypsin (AAT) is another acute phase protein and an archetype member of serpin superfamily, well recognized for its role as a serine protease inhibitor, but also known as an inhibitor of caspase 3, anti-apoptotic and immunomodulatory protein *in vitro* and *in vivo*^11^. Various studies provide evidence that higher AAT levels are associated with cancer and a poor prognosis^12,13^. AAT seems to be directly involved in the distant metastasis of various cancer types, including ovarian, cervical, colorectal, breast, and lung adenocarcinomas^14,15^. Some investigators suggest that AAT can be useful factor for monitoring cancer progression and a person’s response to treatment^16,17^. In line, our current data imply that *SERPINA1* gene encoding AAT and levels of AAT protein have a high influence on lung cancer patient’s survival prognoses^18^.

Actually, a recent study based on 1585 cases with severe inherited AAT deficiency from Sweden National Register and 5999 population-based controls found that death due to cancer is significantly lower in the AAT deficiency carriers than in the controls having normal genetic variant of AAT^19^. This finding is of high interest, since AAT is very important anti-protease in the lungs, and persons with severe inherited AAT deficiency, especially smokers, have an increased risk of developing early-onset obstructive lung disease with emphysema^20,21^. Despite the fact that lung cancer is linked with airflow obstruction and emphysema^22^, AAT deficiency carriers seem not to be at higher risk of developing cancer. This fact further supports existence of undiscovered roles of AAT in tumorigenesis.

Non-small cell lung cancer (NSCLC) accounts for the majority of all lung cancers and has a very poor prognosis. The NSCLC is quite often complicated by pulmonary infections, which impair the therapy and prognosis^23^. Lipopolysaccharides (LPS) are the major pathogenic factors of gram-negative bacteria, mostly seen in lung cancer patients^24^. Experimental and clinical studies report that LPS promotes the growth and metastatic properties of cell lines and primary lung cancer cells from patients^25^. The activation of toll-like receptor 4 (TLR4) signalling is suggested as a key mechanism of gram-negative bacteria in lung cancer progression. Another important signalling mediator is a signal transducer and activator of transcription 3 (STAT3) that is persistently activated in about 50% of NSCLC primary cancers and lung cancer–derived cell lines like H1975^26^. Moreover, LPS is a strong inducer of IL-6, a main cytokine responsible for the induction of AAT synthesis in human cells^27^. Thus, LPS-triggered expression of IL-6 and AAT may actually help cancer cells to escape apoptosis and/or to increase proliferation. Hence, better understanding of the relationship between AAT, inflammation and cancer cell resistance to apoptotic death is of great clinical relevance.

In this study, we aimed to investigate the effects of human AAT on NSCLC apoptosis with and without presence of LPS, as a pro-inflammatory agent. We selected two cell lines strongly differing in the baseline of *SERPINA1* gene (encoding AAT protein) expression, namely H1975 (high *SERPINA1* expression) and H661 (very low *SERPINA1* expression). Our results show that exogenous AAT favours tumour cell growth and inhibits staurosporine (STS)-induced apoptosis and autophagy independently of LPS. Furthermore, in H1975 cells, AAT mediates LPS-induced expression of IL-6, a cytokine related to cancer progression.

## Results

### Supplementation of medium with AAT exaggerates H1975 and H661 cell proliferation

**Figure 1:**
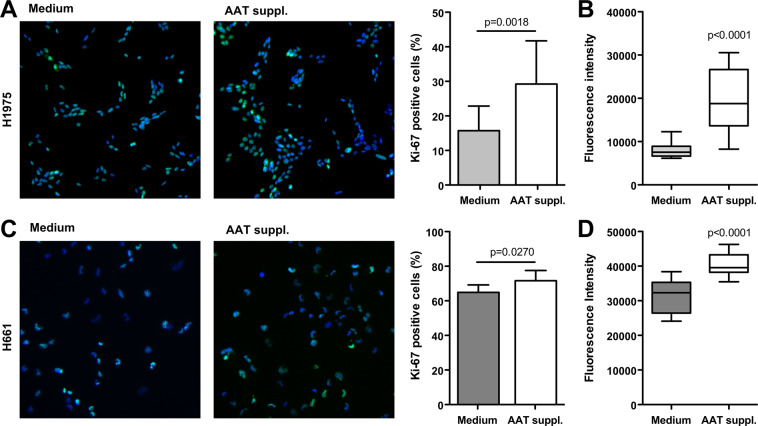
H1975 and H661 cells cultured in complete medium supplemented with 2 mg/ml AAT for 3 weeks show increased proliferation as compared to cells cultured in regular medium. All experimental data were generated from two independent cell cultures of H1975 and H661 cells cultured twice in complete medium without or with supplementation with AAT for 3 weeks. (**A**) (H1975) and C (H661) cells stained with the proliferation marker Ki-67 (*green*) and DAPI (*blue*). Graphs show percentages of Ki-67 positive cells (counted in six fields of three specimens from three independent experiments). (**B**) (H1975) and D (H661) cell proliferation determined with CyQUANT NF assay. Box plots show mean (standard deviation, SD) from three independent experiments carried out in quadruplicates (B) and from two independent experiments carried out with six repeats (D). p < 0.05 was considered as significant.

**Figure 2:**
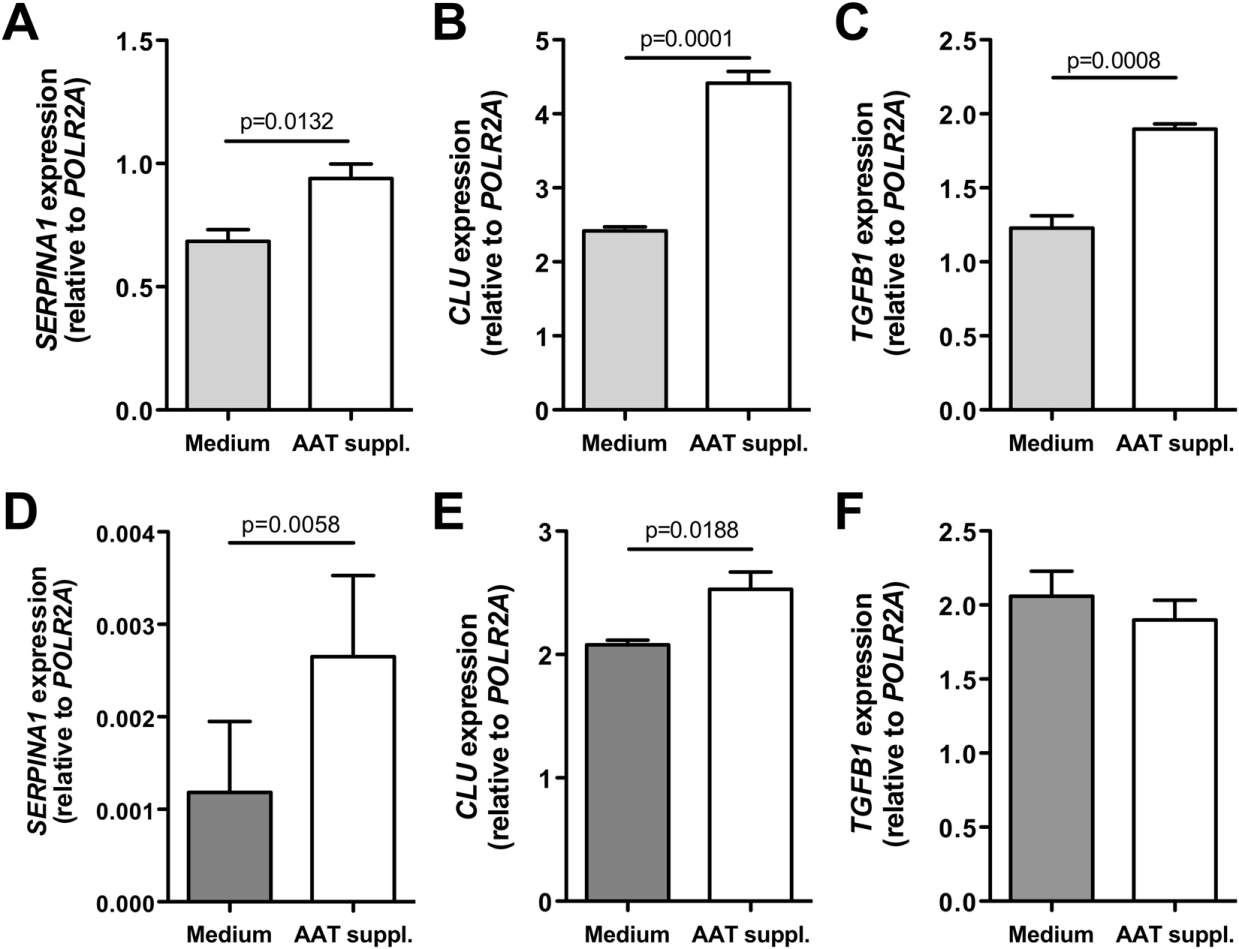
Gene expression of H1975 and H661 cells cultured in medium supplemented with 2 mg/ml AAT for 3 weeks as compared to cells cultured in regular medium. All experimental data were generated from two independent cell cultures of H1975 and H661 cells cultured twice in complete medium without or with supplementation with AAT for 3 weeks. (**A**–**C**) (H1975 cells) and (**D**–**F**) (H661 cells) the expression of *SERPINA1*, *CLU* and *TGFB1* relative to housekeeping gene (*POLR2A*) determined by TaqMan gene expression assays. Bars indicate mean (SD) of three repeats from independent biological samples. p < 0.05 was considered as significant.

Based on our previous finding that higher plasma AAT levels correlate with a poor survival of NSCLC patients^18^, we investigated whether higher levels of AAT in the microenvironment of cancer cells influence them. We cultured H1975 and H661 cells for 3 weeks in a regular medium without and with AAT (2 mg/ml) supplementation. The impact of the longer-term exposure to AAT on the cell proliferation was investigated by using immunofluorescence staining with the proliferation marker Ki-67. As illustrated in Fig. 1A, H1975 cultured in medium supplemented with AAT almost doubled proliferative activity (p = 0.0018) relative to cells grown in a regular medium. This finding was further confirmed by using the fluorescence-based CyQUANT NF assay (Fig. 1B). In H661 cells, effect of AAT supplementations was also significant but less pronounced (Fig. 1C,D). In concordance, both H1975 and H661 cells grown in AAT supplemented medium showed significantly higher expression of *SERPINA1* and *CLU* genes than those grown in the regular medium. In H1975 cells the *TGFB1* gene was also upregulated (58%, p = 0.0001) (Fig. 2A–F).

### Supplementation of medium with AAT improves H1975 and H661 cells resistance against STS-induced apoptosis

**Figure 3:**
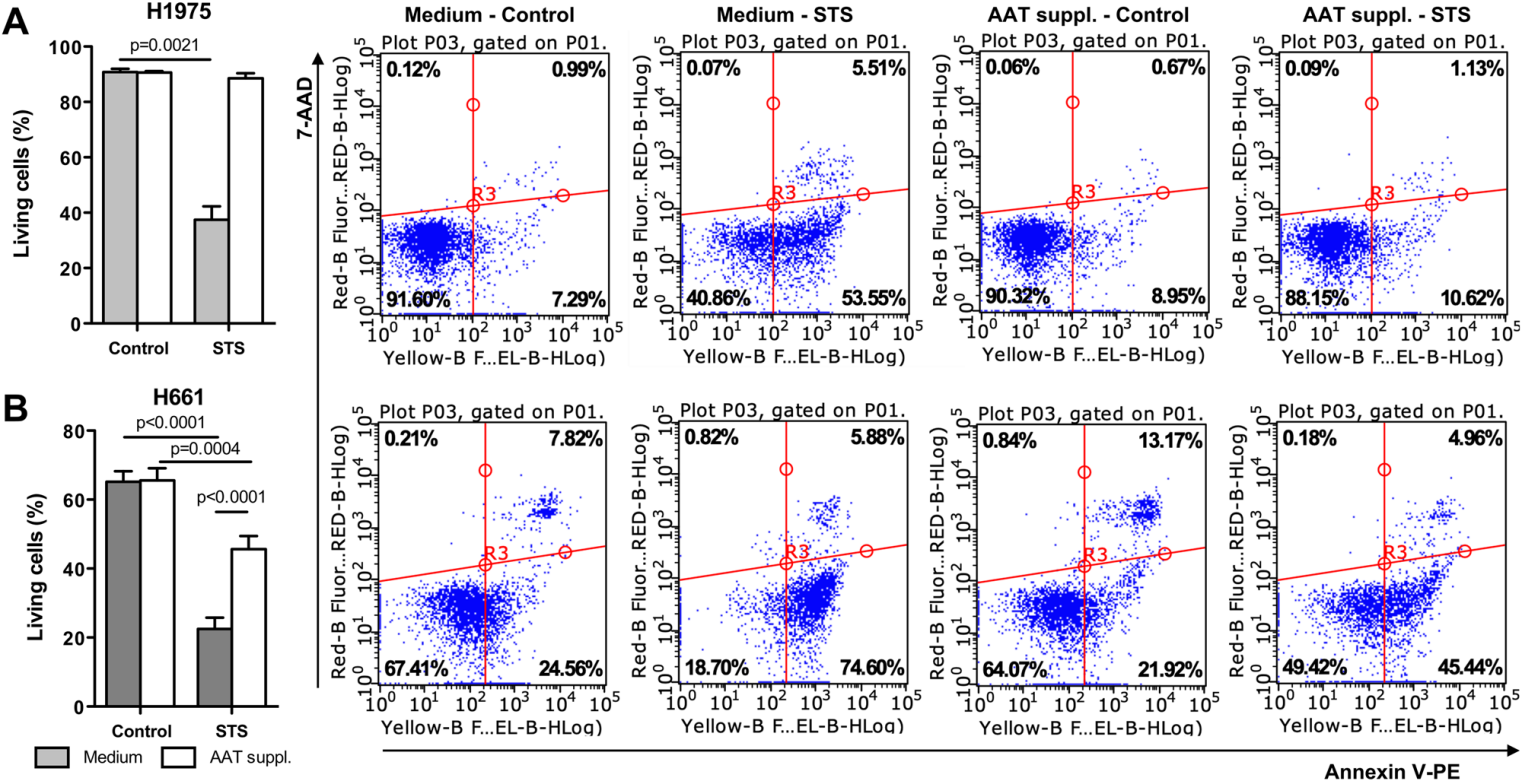
H1975 and H661 cells cultured in medium supplemented with 2 mg/ml AAT for 3 weeks show increased resistance to staurosporine (STS)-induced apoptosis. All experimental data were generated from two independent cell cultures of H1975 and H661 cells cultured twice in complete medium without or with supplementation with AAT for 3 weeks. (**A**) (H1975) and (**B**) (H661) cell supernatants were removed and 50 nM STS was added in serum free RPMI for 18 h. Apoptosis was measured by flow cytometry using PE Annexin V Apoptosis assay. In the bottom left quadrant shown cells classified as “living”. Cells cultured in regular medium (*grey* bars) and in medium supplemented with AAT (*clear* bars). Each bar represents mean (SD) of three repeats from independent experiments. p < 0.05 was considered as significant.

The upregulation of *CLU* and *TGFB1* genes is associated with enhanced cancer cell proliferation and anti-apoptotic properties^28–30^. We therefore investigate if long-term exposure to AAT affects cancer cell sensitivity to staurosporine (STS)-induced apoptosis. For this, the supernatants from the cells cultured with and without AAT were totally removed, and when cells were cultured for 18 h in serum-free medium containing STS (50 nM). Flow cytometry measurements with annexin V/7-AAD double staining revealed substantially higher resistance against STS-induced apoptosis of H1975 and H661 cells cultured with than without AAT (Fig. 3A,B).

### Inhibition of STS-induced apoptosis by AAT does not require a direct STS-AAT interaction and is not affected by LPS

**Figure 4:**
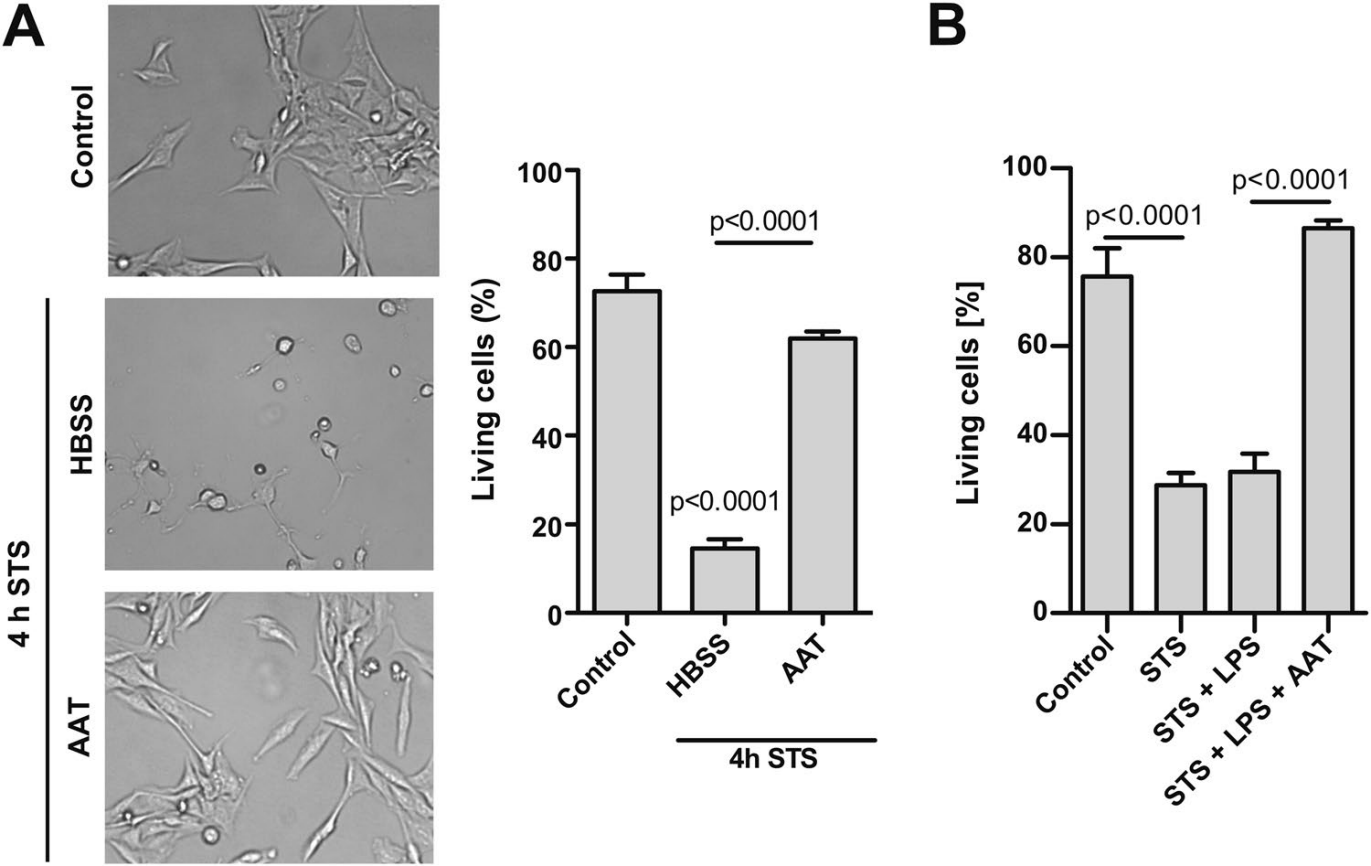
Inhibition of STS-induced apoptosis by AAT does not require a direct STS-AAT interaction and is not affected by LPS. (**A**) Cells were incubated with 50 nM STS for 4 h, thereafter STS was removed and the cells were incubated with HBSS (control) or 1.5 mg/ml AAT in fresh medium for additional 18 h. Cell images were taken under the phase contract microscopy. Apoptosis was analyzed by flow cytometry using PE Annexin V Apoptosis assay to detect living cells. Each bar represents mean (SD) percentage (%) of living cells of three independent experiments. p < 0.05 was considered as significant. (**B**) Cells were treated with STS (50 nM), or LPS (10 µg/ml), or AAT (1.5 mg/ml) separately or in combinations for 18 h. Apoptosis was analyzed by flow cytometry using PE Annexin V Apoptosis assay to detect living cells. Each bar represents mean (SD) percentage (%) of living cells of three independent experiments. p < 0.05 was considered as significant.

In the next set of experiments we directly treated H1975 cells with STS (50 nM) and AAT (1.5 mg/ml) either together or separately in the presence of 10 µg/ml of LPS, and cultured in the serum-free medium for 18 h. The purpose of these experiments was to exclude the possibility that AAT may directly bind STS and eliminate its pro-apoptosis effects, and to examine whether LPS affects antagonizing properties of AAT on STS-induced cell apoptosis. We pre-incubated H1975 cells with STS for 4 h and completely removed supernatants containing STS. Afterwards, we cultured cells in a serum-free medium without or with additions of AAT (1.5 mg/ml) for 18 h. Results showed that in the presence of AAT cells preserved morphology and were strongly resistant to STS-induced apoptosis (Fig. 4A). As presented in Fig. 4B, LPS does not affect STS-induced apoptosis and inhibitory effects of AAT. This latter finding was also reproduced in H661 cells (data not shown).

### AAT does not inhibit chloroquine (CQ) or streptonigrin (STN)-induced apoptosis independent of the presence of LPS

**Figure 5:**
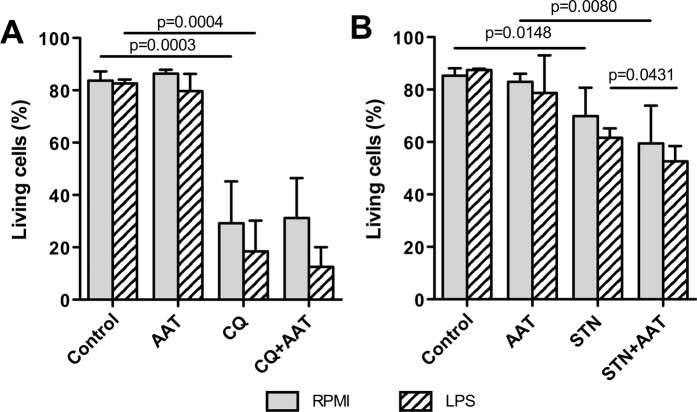
Exogenously added AAT does not interfere with chloroquine (CQ) or streptonigrin (STN)-induced apoptosis in H1975 cells independent on the presence of LPS. Cells were treated with 200 µM CQ or 200 nM STN alone or together with AAT (1.5 mg/ml) and LPS (10 µg/ml) for 18 h. Percentages of living cells were determined by flow cytometry using PE Annexin V Apoptosis assay. Each bar represents mean (SD) of three independent experiments. p < 0.05 was considered as significant.

We also tested whether AAT specifically attenuates STS-induced apoptosis or acts as a broader anti-apoptotic agent. For this, H1975 cells we treated with 200 µM CQ or 200 nM STN alone or in the presence of AAT (1.5 mg/ml) in serum-free medium for 18 h. When compared to non-treated controls or cells treated with AAT, the percentage of living cells decreased significantly under CQ or STN treatment. Unfortunately, CQ and STN-induced cancer cell death was not inhibited by AAT (Fig. 5A,B). Similar experiments performed in the presence of LPS showed no significant changes in the CQ, STN or AAT behaviours (Fig. 5A,B, *hatched bars in the graph*). These results indicate that independently of the presence of inflammatory stimuli, such as LPS, AAT inhibits only certain pathways for apoptosis induction.

### AAT prevents STS-induced cleavage of pro-caspase 3 and a suppression of AKT/MAPK pathways

**Figure 6:**
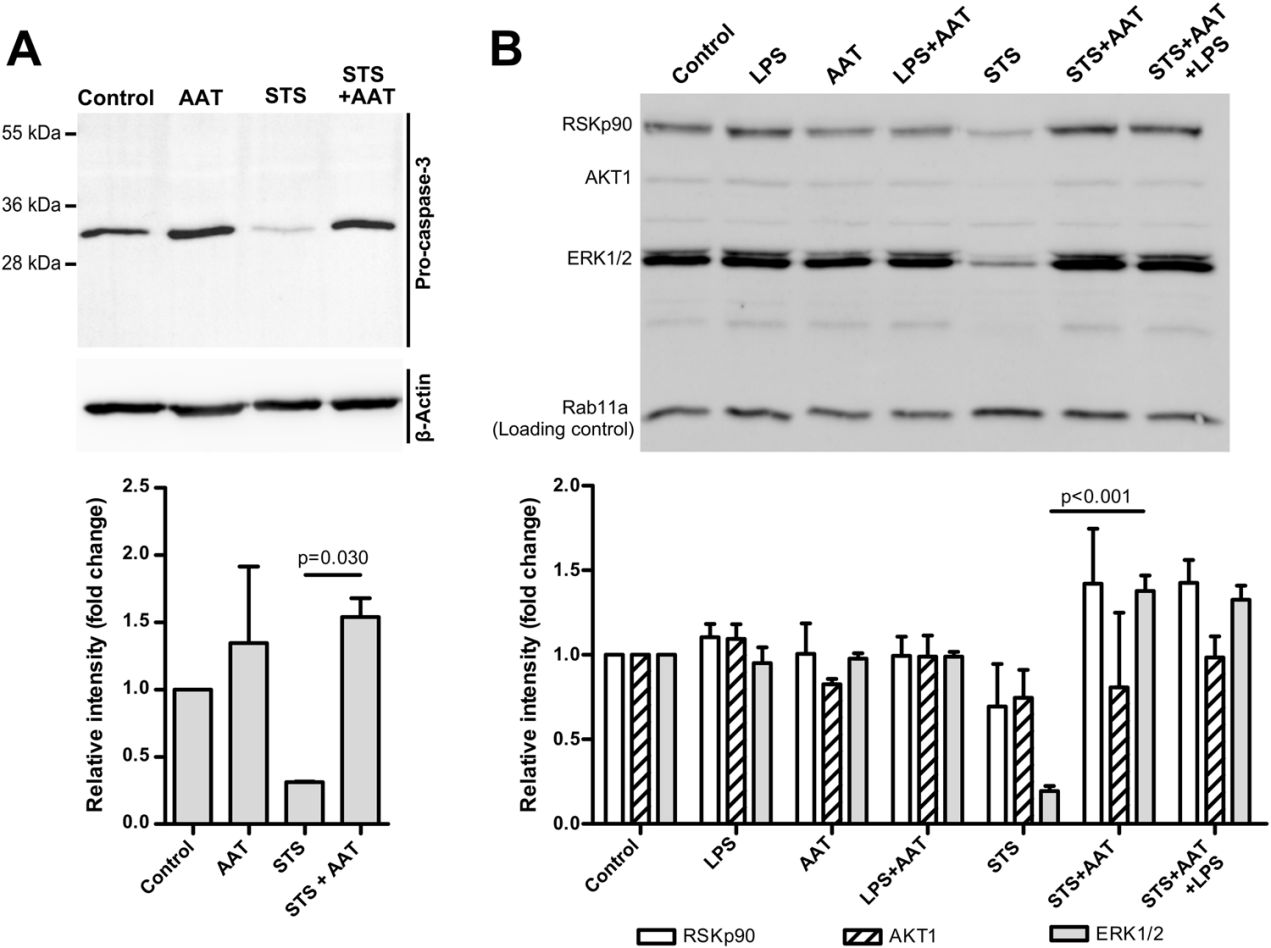
Exogenously added AAT prevents STS-induced pro-caspase-3 cleavage and suppression of Akt and MAPK pathways. (**A**) H1975 cells were cultured for 18 h in the medium alone (controls), and in the medium containing 50 nM STS alone or in combination with 1.5 mg/ml AAT. Cell lysates were applied to Western blot analysis. β-actin was used as a loading control. Fold changes were calculated for each band using the ratio relative to β-actin, as a loading control, and then normalized by the experimental control. Bars represent mean (SD) of three independent experiments. (**B**) H1975 cells were incubated for 15 min in medium alone (controls) and in medium containing 50 nM STS, 10 µg/ml LPS, or 1.5 mg/ml AAT alone or in combinations. Cell lysates were applied to Western blot and analysed with AKT/MAPK signalling pathway antibody cocktail targeting AKT1 (phospho S473), ERK1/2 (phospho Y204/197), RP-S6 (phospho S235/236) and p90 RSK (phospho S380). Fold changes were calculated for each band using the ratio relative to Rab11a, as a loading control, and then normalized by the experimental control. Bars represent mean (standard error, SEM) from three independent experiments. Representative blots are shown. p < 0.05 was considered as significant.

Previous studies have shown that STS mediates cell death via activation of caspase-3 and the down-regulation of the PI3/Akt and MAPK/Erk pathways, which are important for cancer cell survival^31^. Western Blot analysis of H1975 cell lysates prepared after cell treatment with STS for 18 h showed a clear reduction (by about 69%) in the levels of pro-caspase-3 (32 kDa protein) (Fig. 6A). This down-regulating effect of STS on pro-caspase 3 was abolished in the presence of AAT. Noticeably, AAT alone slightly induced (by about 26%) pro-caspase-3 protein levels if compared to non-treated control cells. Furthermore, H1975 cells exposed to STS for 15 min showed a marked reduction in the phosphorylation of PI3K downstream effectors, such as AKT1 (phospho S473), and the MEK downstream effectors, such as ERK1/2 (phospho Y204/197) and p90RSK (phospho S380), as compared to non-treated control or LPS, AAT or LPS/AAT treated cells. In the presence of AAT, all blocking effects of STS on AKT/MAPK pathways were lost. We also show that LPS alone or used together with AAT or STS plus AAT, has no effect (Fig. 6B). These data are consistent with a function of AAT as an inhibitor of caspase-3^32^.

### AAT prevents autophagy activation by STS

**Figure 7:**
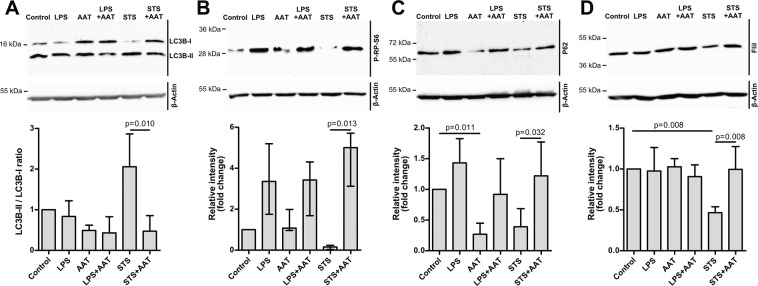
Exogenously added AAT prevents STS-induced inhibition of autophagy. H1975 cells were cultured in medium alone (controls) or in medium containing 50 nM STS, 10 µg/ml LPS, or 1.5 mg/ml AAT separately and in combination for 2 or 18 h. Cell lysates were analysed by Western blot. (**A**) for LC3B analysis, cell lysates were prepared after 2 h incubation time. LC3B-II versus LC3B-I ratios were calculated. Bars represent mean (SD) of three independent experiments. (**B**) for p-RB-S6 analysis, cell lysates were prepared after 2 h incubation time in lysis buffer containing phosphatase inhibitory cocktail. Bars represent median (25–75 percentiles) of four independent experiments. (**C**) for p62 analysis, cell lysates were prepared after 2 h incubation time. Bars represent mean (SD) of six independent experiments. (**D**) FliI was examined after 18 h of cell incubation. Bars represent mean (SD) of four independent experiments. Fold changes were calculated for each band using the ratio relative to β-actin, as a loading control, and then normalized by the experimental control. Representative blots are shown. p < 0.05 was considered as significant.

Several lines of evidence suggest that autophagy activation by the apoptotic process is a critical step for cell death^33,34^. Based on the findings that AAT protects H1975 cells against STS-induced apoptosis, we have speculated that AAT may also abrogate STS effects on autophagy. Therefore, we examined the levels of LC3, sequestosome-1 (SQSTM/p62) and Flightless-I (FliI) as well as the activity of mTOR (phosphorylation of mTOR substrate RP-S6) - all involved in the autophagy process. As shown in Fig. 7A–C, the exposure of H1975 cells to STS (50 nM) for 2 h resulted in an increased LC-II/LC-I ratio (about 2-fold) and a strong decrease in the levels of phospho RP-S6 (p-PR-S6) and p62 protein. Furthermore, the levels of the flightless-I protein (FliI), acting as a negative regulator for p62-mediated autophagy, were decreased in response to STS (Fig. 7D). However, when cells were co-treated with STS and AAT (1.5 mg/ml), the effects of STS on LC-II/LC-I ratio and on the p-RP-S6, p62 and FliI proteins were significantly reduced. Notably, in cells treated with STS and AAT together, p-RP-S6 levels increased markedly relative to STS treated cells as well as relative to non-treated controls (Fig. 7B). In the same experimental setting LPS and AAT used separately or together, had no significant effect on LC-II/LC-I ratio and FliI levels, whereas LPS alone or in the presence of AAT increased levels of p-RP-S6 protein. Unexpectedly, AAT alone reduced p62 protein levels as compared to non-treated controls whereas LPS abrogated this suppressive effect of AAT. This phenomenon we also observed after cell culture for 18 h (Supplementary Fig. 1). Altogether, our results show that STS-induced apoptosis and autophagy can be suppressed by AAT.

### AAT abolishes inhibitory effect of STS on cancer cell migration

**Figure 8:**
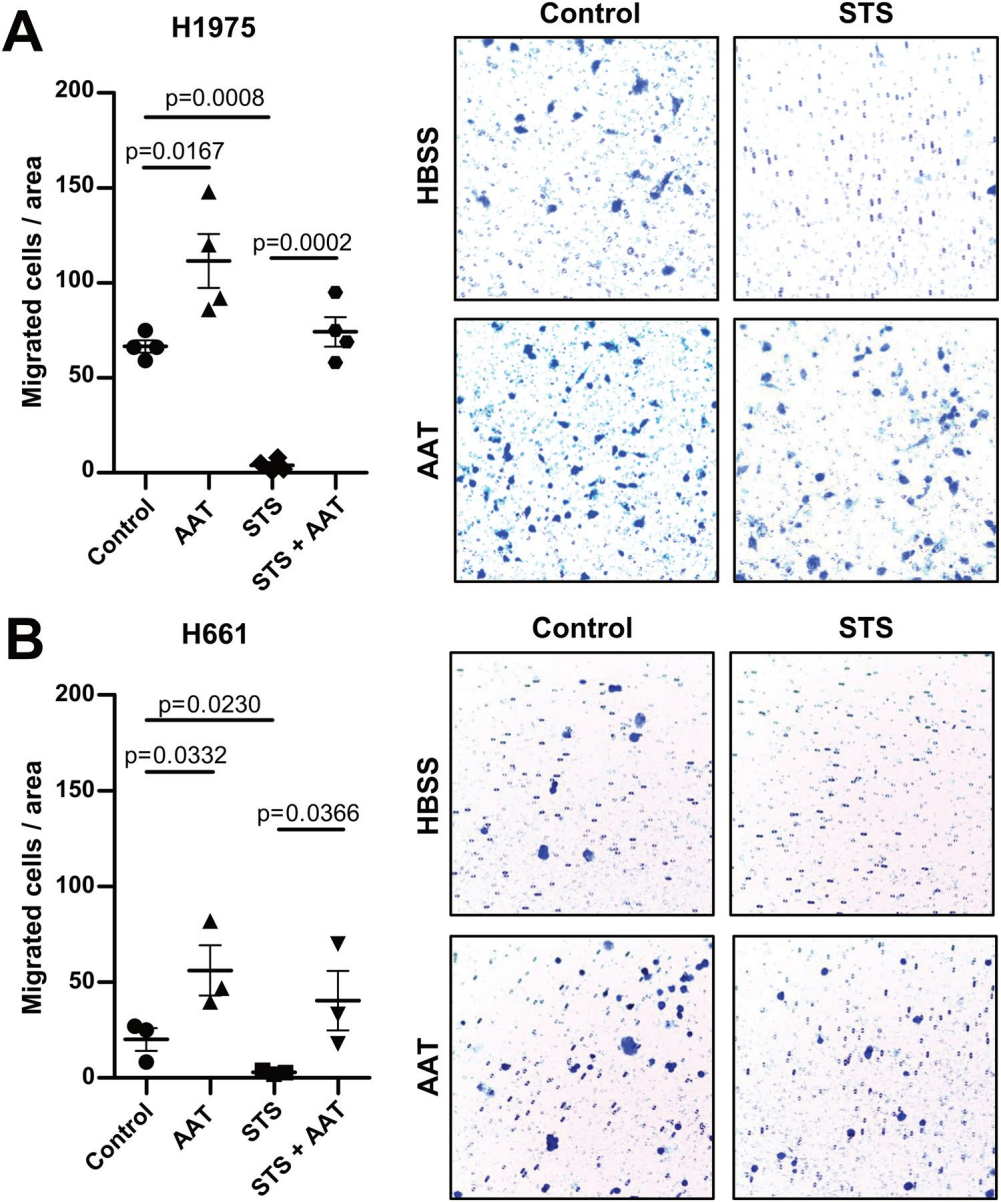
Exogenously added AAT prevents STS-induced loss of cancer cell migration. H1975 and H661 cells migration was studied within 3 h with addition to the medium HBSS (controls), 50 nM STS or 1.5 mg/ml AAT separately or in combination. The migrated cells were stained with 0.1% crystal violet. For quantification, the stained cells were counted under a microscope (Leica DM750) with 10x objective from six areas. (**A**) (H1975 cells), the graph indicates data from four independent experiments as mean (SD) and the individual values. (**B**) (H661 cells), the graph shows data from three independent experiments as mean (SD) and the individual values. p < 0.05 was considered as significant.

To demonstrate that the observed STS-damaging effects in cancer cells can be blocked in the presence of AAT, we analysed cell migration. In the presence of AAT, H1975 cells showed significantly enhanced (by about 68%, p = 0.0167) migratory properties compared to the untreated cells. As expected, STS (50 nM) drastically reduced (by about 94%, p = 0.0002) the number of migrated cells whereas this effect of STS was totally lost in the presence of AAT (Fig. 8A). Similarly, the H661 cells showed 2-fold improved migratory properties in the presence of AAT whereas STS-treated cells completely lost their ability to migrate (Fig. 8B). Again, AAT abolished the inhibitory effect of STS on cell migration and even cell migration we found to be elevated relative to untreated (control) cells – the mean value of migrated cells doubled (Fig. 8B).

### STS and LPS show different effects on *SERPINA1* gene and AAT protein expression in cancer cells

**Figure 9:**
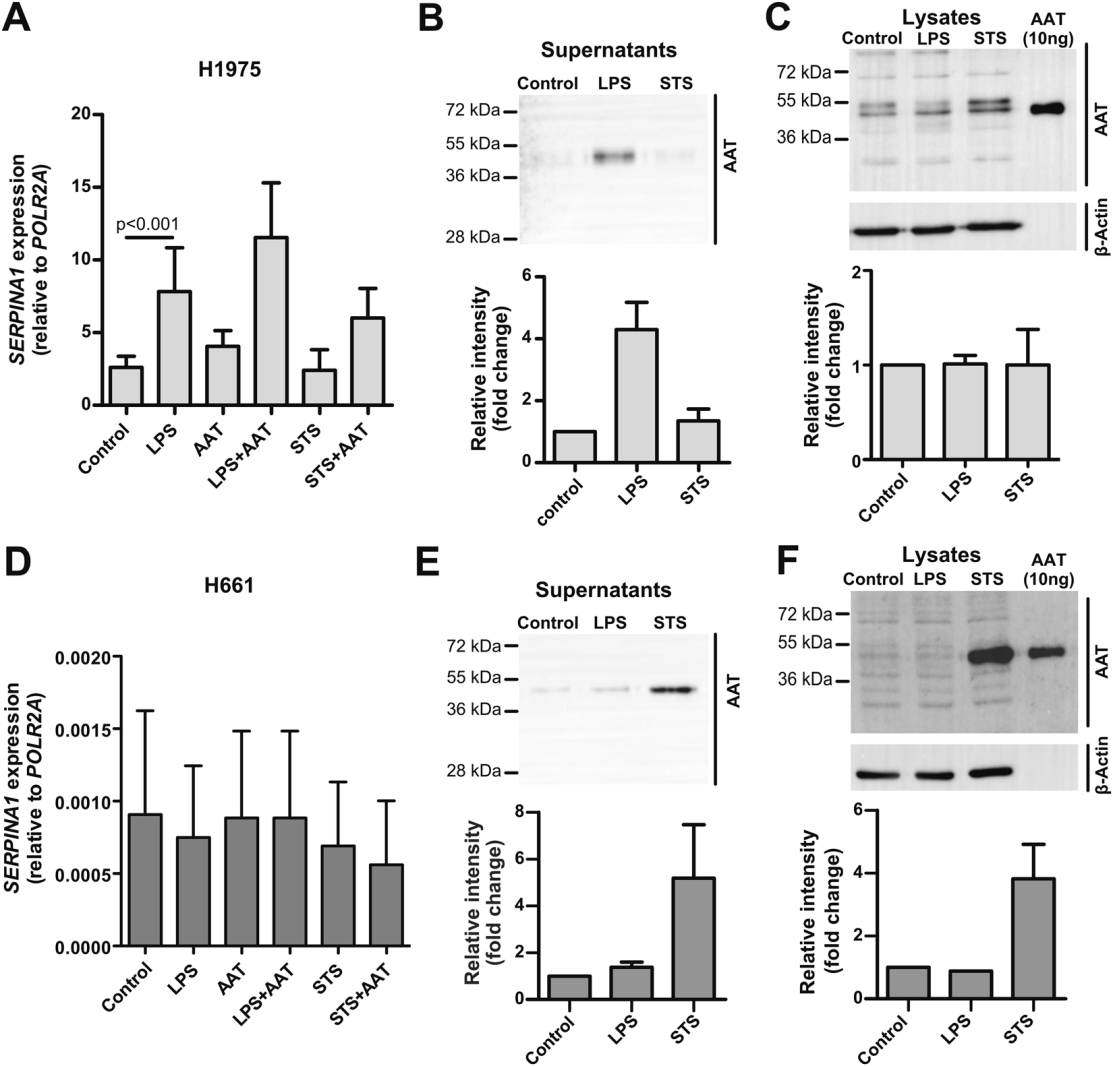
Cell specific effects of STS and LPS on *SERPINA1* gene expression and AAT protein. (**A**) (H1975 cells) and (**D**) (H661 cells) were cultured for 18 h alone or in the presence of 10 µg/ml LPS, 50 nM STS, or 1.5 mg/ml AAT separately and in combinations. Graphs show *SERPINA1* gene expression relative to housekeeping gene *POLR2A*. Bars represent mean (SD) of three independent experiments. p < 0.05 was considered as significant. (**B**,**C**) H1975 cells were cultured for 18 h in the medium containing HBSS (controls), 10 µg/ml LPS or 50 nM STS. Supernatants and cell lysates were analysed by Western blot. β-actin was used for loading control. Representative blots are shown out of three independent experiments. Densitometric data presented as mean (SD) of four (**B**) and three (**C**) independent experiments for supernatants and for lysates. 10 ng AAT was added to the blot as a positive control. (**E,F**) H661 cells were cultured for 18 h in the medium containing HBSS (controls), 10 µg/ml LPS, or 50 nM STS. Supernatants and cell lysates were analysed by Western blot. Representative blots are shown out of three independent experiments. Densitometric data presented as mean (SD) of three independent experiments for supernatants and two for lysates. 10 ng AAT was added to the blot as a positive control.

We next asked whether our treatments alter AAT synthesis in cancer cells. As illustrated in Fig. 9, the exposure of H1975 cells for 18 h to LPS resulted in an induction of *SERPINA1* gene (by 2.5-fold, p < 0.001) while STS had no effect. When these cells were treated with LPS in the presence of exogenous AAT, *SERPINA1* expression further raised by about 48% as compared to LPS treated cells (Fig. 9A). Opposite, neither LPS nor STS alone or used together with AAT affected very low *SERPINA1* expression in H661 cells (Fig. 9D).

To further validate LPS and STS effects on AAT protein levels, we analysed cell culture supernatants and cell lysates by western blotting. As shown in Fig. 9B,C, in H1975 cells LPS but not STS induced AAT protein release, which paralleled with increase in *SERPINA1* gene expression. Although STS had no significant effect on *SERPINA1* gene in H661 cells, it exhibited a capacity to induce AAT protein levels (Fig. 9E,F). This unexpected finding that STS-induces AAT protein production, at least in part, may explain why addition of AAT to STS-treated H661 cells not only restored but also enhanced migratory properties (Fig. 8B). In H661 cells, LPS had no effect on AAT protein levels.

### AAT increases LPS effect on IL-6 expression and release in H1975 cells

**Figure 10:**
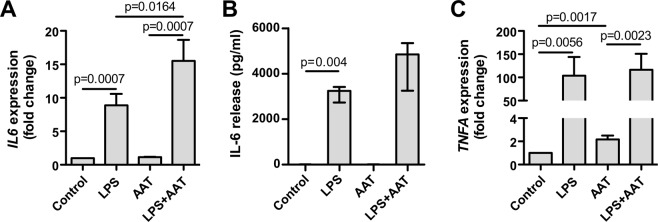
Effects of AAT, LPS or their combination on IL-6 and TNFα in H1975 cells. Cells were cultured for 18 h in the medium containing HBSS (controls), 10 µg/ml LPS or 1.5 mg/ml AAT separately and in combination. (**A**) *IL6* gene expression relative to housekeeping gene *POLR2A* and normalized to controls (fold change). Each bar represents mean (SD) of three independent experiments. (**B**) IL-6 release measured by ELISA. Each bar represents median (25–75 percentile) of eight independent experiments. (**C**) *TNFA* gene expression relative to housekeeping gene *POLR2A* and normalized to controls (fold change). Each bar represents mean (SD) of three independent experiments. p < 0.05 was considered as significant.

Because H661 cells during treatment with LPS show no response in *SERPINA1* expression and have extremely low *IL6* gene expression relative to *POLR2A* [(mean (SD), 0.0012 (0.0002), n = 3 independent experiments] we performed following experiments only with H1975 cells. Thus, when compared to non-treated controls or H1975 cells treated only with AAT, in response to LPS these cells strongly increased IL-6 expression (9-fold, p = 0.0007) and release (p = 0.004), and *TNFA* gene expression (100-fold, p = 0.0056). Although AAT by itself had no effect on *IL6* and *TNFA* genes, it magnified LPS-induced *IL6* gene expression and IL-6 protein release (Fig. 10A,B). This latter result may also explain the enhanced *SERPINA1* gene expression in H1975 cells treated with LPS/AAT versus LPS alone (Fig. 9A). For LPS-induced *TNFA* expression, though, we observed no additive effect of AAT (Fig. 10C).

### AAT induces TLR4 levels in H1975 cells

**Figure 11:**
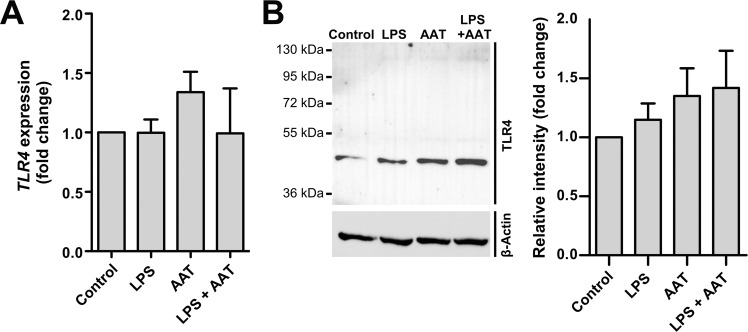
Effect of exogenously added LPS and AAT on *TLR4* gene and TLR4 protein levels in H1975 cells. Cells were cultured for 18 h in the medium containing HBSS (controls), 10 µg/ml LPS, 50 nM STS, or 1.5 mg/ml AAT separately and in combinations. (**A**) *TLR4* gene expression was related to housekeeping gene *POLR2A*. Results are shown as fold changes normalized to non-treated controls. Each bar represents mean (SD) of three independent experiments. (**B**) TLR4 protein levels were analyzed by Western blot. Fold changes were calculated for each band using the ratio relative to β-actin, as a loading control, and then normalized by the experimental control. Each bar represents mean (SD). Representative blot is shown out of six independent experiments.

It is well-known that LPS activates TLR4 dependent pathway to increase inflammatory cytokines like IL-6^35^. LPS showed no significant effect on *TLR4* expression and protein levels in H1975 cells. However, *TLR4* expression increased by about 34% in the presence of AAT relative to LPS treated cells and non-treated controls (Fig. 11A). Likewise, a slight but constant increase in TLR4 protein levels was observed in AAT and AAT/LPS treated cells (Fig. 11B). It is important to notify that in these cancer cells a specific band of TLR4 protein was lacking full length.

### Stattic, a specific STAT3 inhibitor, lowers LPS and AAT/LPS-induced IL-6 expression and release

**Figure 12:**
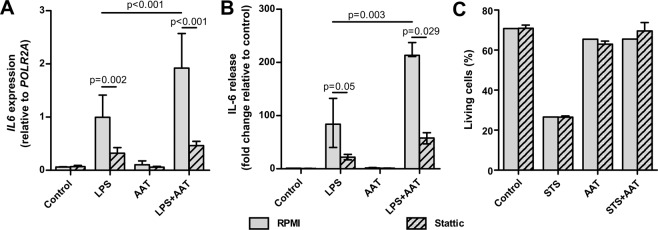
Effects of stattic (STAT3 inhibitor) on LPS-induced IL-6 production and STS-induced apoptosis in H1975. Cells were treated with 5 µM stattic for one hour prior to addition of HBSS (control), 10 µg/ml LPS, or 1.5 mg/ml AAT separately and in combination for 18 h. (**A**) *IL6* gene expression was related to housekeeping gene *POLR2A*. Each bar represents mean (SD) from six independent experiments. (**B**) Supernatants were analysed for IL-6 release by ELISA. Each bar represents median (25–75 percentile) of four independent experiments. (**C**) Apoptosis was analyzed by flow cytometry using PE Annexin V Apoptosis assay to detect living cells. Each bar represents mean (SD) of living cells (percentage) from two independent experiments for stattic. p < 0.05 was considered as significant.

According to previous studies, STAT3 specifically modulates LPS-driven IL-6 production^36^. Indeed, in the presence of 20 µM stattic, the effects of LPS and LPS plus AAT on *IL6* gene expression in H1975 cells significantly decreased (Fig. 12A). In line with the results presented in Fig. 10, the expression of *IL6* gene was higher in AAT/LPS versus LPS treated cells. As presented in Fig. 12B, stattic also strongly suppressed IL-6 release in both LPS and LPS/AAT treated cells. Again, the levels of IL-6 protein were significantly higher in supernatants collected from AAT/LPS as compared to LPS treated cells. Noticeably, stattic did not show toxicity in H1975 cells and did not influence AAT property to inhibit STS-induced apoptosis (Fig. 12C). These findings suggest that in H1975 cells, STAT3 activity is necessary for the induction of IL-6 by LPS and LPS/AAT but it is not essential for the ability of AAT protein to inhibit STS-induced apoptosis.

## Discussion

Cancer cell resistance to anticancer drugs is mediated by various factors, specifically inhibitors of cell apoptosis. Among these are serine protease inhibitors (serpins) representing up to 2–10% of circulating plasma proteins. The serpins regulate many biological reactions such as coagulation, complement and inflammation, angiogenesis and apoptosis. Certain human serpins are associated with anti-cancer roles^37^ while others with cancer and metastasis-promoting functions^38^. Increased circulating and tumor-associated levels of specific serpins have been associated with poor outcome in different types of cancer. The increased levels of AAT, one of the most abundant serpins in human circulation (1–2 g/L), have also been associated with worse overall patient survival and with more advanced tumor stages. In accordance, in a cohort of 351 NSCLC patients, we recently reported that higher circulating levels of AAT are prognostic for the patient’s worse outcome^18^.

Although AAT is best characterized as an inhibitor of neutrophil elastase, it also inhibits caspase-3 activity, and was reported to inhibit staurosporine (STS)-triggered neutrophil apoptosis^32,39^. STS is a microbial alkaloid possessing anti-proliferative and pro-apoptotic activity in several human cancer cell lines. At least one way by which STS induces apoptosis is the activation of caspase-3^40^. Based on this knowledge, we raised a hypothesis that cancer cells may employ anti-apoptotic properties of AAT to evade STS-induced apoptosis. We first aimed to investigate the properties of NSCLC after long-term culture in the medium supplemented with AAT. Because circulating AAT increases rapidly to concentrations of 2 mg/ml and more in response to inflammation and cancer, we chose to supplement cell culture medium with 2 mg/ml of AAT. Two cell lines H1975 and H661 we cultured for three weeks either in a regular medium or in the medium supplemented with AAT. The comparison between these two cell culture conditions clearly showed that the cells grown in the presence of AAT have better proliferative properties, and higher expression of *SERPINA1* and *CLU* genes. Specifically in H1975 cells, we observed a marked increase in expression of *TGFB1*, a known inducer of clusterin expression in cancer cells^41^. The clusterin (apolipoprotein J) exhibits pro-tumorigenic, and anti-apoptotic activities and plays an important role in tumor invasiveness. In fact, serum levels of clusterin may potentially be useful as a diagnostic biomarker of NSCLC^42,43^.

Apoptosis has a high impact on the development of tumors as well as on their response to therapy^44^. Actually, both cells lines cultured in medium supplemented with AAT exhibited significantly enhanced resistance towards STS-induced apoptosis if compared to those cells cultured in a regular medium. Similarly, AAT added with STS simultaneously or added to the STS-pre-treated cells, exerted a strong anti-apoptotic effect. Previous finding that higher circulating levels of AAT in NSCLC patients correlate with worse prognosis together with our current data provide stronger evidence that in the presence of AAT cancer cells can improve their pro-tumorigenic characteristics and resistance to apoptosis.

Since AAT is an acute phase protein regulated by inflammation, we wanted to investigate the pro-tumorigenic properties of AAT in the presence of LPS, a well-known factor of gram-negative bacteria that is often seen in lung cancer patients^45^ and worsen patients prognosis^46^. Moreover, LPS induces IL-6^47^ a cytokine that specifically mediates expression and secretion of AAT^48^. Whether LPS influences pro-tumorigenic activities of AAT in lung cancer cells remains unknown. Therefore, in the following experiments we aimed to determine the putative influence of LPS on the ability of AAT to counteract apoptosis and/or to enhance tumor-promoting properties of cancer cells. All experiments we performed with 10 μg/ml LPS since this concentration induced IL-6 and AAT production in H1975 cells and showed no effects on cell viability.

As mentioned above, STS regulates cell death through activation of caspase-3 that is also essential to autophagic activity^49,50^. According to previous findings, STS-mediated activation of caspase-3 occurs through pro-caspase 3 cleavage and the suppression of the AKT/MAPK pathways^51^. In line, STS significantly reduced pro-caspase-3 levels and suppressed AKT/MAPK pathways in H1975 cells as determined by a marked reduction in the phosphorylation of AKT1, ERK1/2 and p90RSK. The STS also suppressed the FliI, an actin remodelling protein that enhances tumour progression by decreasing apoptosis and inducing cell invasion. However, in the presence of AAT, STS failed to stimulate pro-apoptotic pathways. At the concentration used, LPS did not show significant influence on AAT and STS behaviours.

In general, the regulation of apoptosis and autophagy is intimately connected; autophagy can inhibit apoptosis but activation of autophagy can also lead to apoptotic cell death^52^. Because STS is shown to regulate cell death through the activation of autophagy, we investigated whether alterations in H1975 cell viability under STS and STS/AAT treatments affect autophagy. Currently LC3 is considered as the best autophagosome marker because the amount of LC3-II reflects the number of autophagosomes. On the other hand, the LC3-II amount as well as LC3-II/LC3-I ratio does not necessarily assess the autophagic activity, since the amount of LC3-II might increase not only due to autophagy activation but also due to the inhibition of autophagosome degradation^53,54^. For monitoring autophagic activity therefore is also used p62, another important marker that directly binds to LC3 and degrades by autophagy. Thus, the activation of autophagic flux relates to a decline in p62 protein expression. Reduced phosphorylation of mTOR also well reflects the activation of autophagic flux^55^. Accordingly, STS significantly induced LC3-II protein but also strongly reduced levels of p62 and phosphorylated mTOR proteins. These results support a notion that STS-triggered H1975 cell apoptosis occurs in parallel to the activation of autophagy. In contrast, in the presence of AAT or AAT with LPS, STS did not induce significant changes in LC3-II and p62 protein levels, phosphorylation of the mTOR substrate RP-S6 and FliI protein levels relative to controls. It seems plausible that, in H1975 cells, AAT is able to prevent STS-induced apoptosis by preventing activation of autophagy, and its effect is independent of LPS.

Of particular interest is that although AAT by itself had no effect on LC3 and FliI protein levels, and RP-S6 phosphorylation, it significantly reduced levels of p62 protein. On the other hand, LPS significantly enhanced the p62 protein level, independently of the presence of AAT. Hypothetically, the downregulation of p62 by AAT might be of importance for acquiring its tumour promoting functions^38^. Reduced p62 levels may be important for tumour survival under hypoxic, metabolic, or other stress situations, such as serum-free conditions used in our experiments. In case of LPS, the induction of p62 may be important for inflammatory cytokines expression^56,57^. At this stage, we couldn’t address this hypothesis and further studies will be conducted.

The finding that AAT efficiently blocks STS-induced cancer cells death raised a question whether AAT can also block other pro-apoptotic compounds employing different cellular pathways than STS. For instance, chloroquine (CQ) is an anti-malarial drug, developed to inhibit autophagy^58^ which also induces apoptosis of cancer cells^59^. Streptonigrin (STN) is an aminoquinone antitumor and antibacterial antibiotic produced by *Streptomyces flocculus* which induces apoptosis through the p53-dependent pathway^60^. Unfortunately, AAT alone as well as in the presence of LPS was not able to protect H1975 cells against CQ or STN-induced death. It remains to be determined whether AAT solely affects the caspase-3-driven cell apoptosis.

Based on the series experiments discussed above, we conclude that LPS does not influence protective effects of AAT on STS-induced H1975 cell apoptosis although LPS upregulates *SERPINA1* expression and AAT protein levels. Previous studies showing that AAT is an inhibitor of pro-inflammatory cytokine - including IL-6 - production^61^ prompted us to investigate effects of exogenously added AAT on LPS induced IL-6 production in H1975 cells. Unexpectedly, however, in the presence of AAT, LPS-induced expression of both IL-6 and AAT was even higher than in LPS alone treated cells. Likewise, relative to control cells, AAT alone and together with LPS, persistently induced TLR4 protein levels. LPS activates TLR4-dependent pathway in order to induce cytokine production^35^, which may explain why AAT enhanced *IL6*, and *SERPINA1* expression in LPS treated cells.

LPS also significantly induced *TNFA* mRNA in H1975 cells however; in this case, AAT did not amplify LPS-effect on TNFα production. The expression of some TLR4-induced genes requires the protein kinase activity of the EGFR^62^. H1975 carry EGFR L858R + T790M mutations^63^, which may determine differences in the regulation of IL-6 and TNFα by AAT/LPS treatments. Moreover, based on mononuclear phagocyte models, investigators reported that the upregulation of TLR4 via IL-6 can increase the responsiveness of cells to LPS whereas TNFα may down-regulate TLR4 leading to LPS hypo reactivity^64^. So far, the effect of AAT on the LPS-induced cytokine production in different NSCLC has not been examined, and therefore further studies are of great importance in this area.

IL-6 is a classic pro-inflammatory cytokine that signals through STAT3 as part of the acute phase response, and acts directly in the prevention of apoptosis^65^. According to a recent study, among several plasma cytokines only IL-6 shows significantly increased expression and a strong association with disease development in patients with NSCLC^66^. Although, LPS is a strong inducer of IL-6 production in H1975 cells, as mentioned above, LPS showed no influence on STS-induced H1975 cells apoptosis and the anti-apoptotic effects of AAT. Stattic, a specific STAT3 inhibitor, diminished LPS-induced IL-6 expression and release in H1975 cells however, despite this, showed no influence on cell viability neither alone nor used together with STS and AAT. Thus, our current results do not support an anti-apoptotic role of IL-6 in H1975 cells.

Altogether, data from this study provide evidence that human AAT can improve lung cancer cell proliferative properties and the resistance against STS-induced death. During an inflammatory response, tissue concentrations of AAT may increase as much as 11-fold because of local synthesis by resident and invading inflammatory cells^67^. As such, AAT exerts cell protective immune-modulatory functions via both -protease inhibitory and non-inhibitory pathways. This ability of AAT may also create an environment that is enriched with tumour-promoting factors and thus favour tumour development. A comprehensive knowledge of the role of AAT in the apoptosis of the cancer cells might help us better understand the relationship between inflammation and cancer development and in long run may also help to improve cancer therapies.

## Materials and methods

### Cell culture and experiments

NSCLC NCI-H661 (ATCC HTB-183) and NCI-H1975 (ATCC CRL-5908) cell lines were purchased from ATCC, the Global Bioresource Center (Virginia, Waltham, MA, USA). Tests for mycoplasma (Hoechst DNA stain, Agar culture, PCR-based assay) were performed by the supplier. Cells were maintained in RPMI 1640 media supplemented with 10% fetal bovine serum (Gibco Thermo Fisher Scientific, Waltham, Massachusetts, USA) at 37 °C and 5% CO_2_. Alternatively, cells were cultures for three weeks in complete medium supplemented with 2 mg/ml AAT protein. Human plasma purified and endotoxin free AAT was prepared in sterile Hank’s buffered salt solution (HBSS, Biochrom, Berlin, Germany) based on the commercial preparation of AAT (Respreeza, CSL Behring, Germany) by ultrafiltration (Amicon Ultra Centrifugal Filters, cut-off of 10 kDa; Merck, Darmstadt, Germany). During experiments, cells were cultured in RPMI without serum for determined time points alone, with 10 µg/ml lipopolysaccharides (LPS) from *Echerichia coli* O55:B5 (Sigma-Aldrich, St. Louis, Missouri, USA), 10 or 50 nM staurosporine (STS, Sigma Aldrich), 200 µM chloroquine diphosphate (CQ, Sigma Aldrich), 200 nM streptonigrin from *Streptomyces flocculus* (STN, Sigma Aldrich), 1.5 mg/ml AAT alone or in combinations. For the control, an appropriate volume of HBSS was added. In some experiments, cells were pre-treated with 50 nM STS in RPMI or RPMI for 4 h. Thereafter, cells were washed once with PBS before RPMI with AAT or HBSS was added for additional 18 h incubation. In some experiments, cells were pre-treated with 5 µM or 20 µM STAT3 inhibitor V stattic (Sigma Aldrich) for 1 h prior to addition of LPS, STS or AAT and a further incubation for 18 h.

### RNA isolation and cDNA preparation

Total RNA was isolated using the RNeasy Mini Kit (Qiagen, Venlo, The Netherlands) following the manufacturer’s instructions. RNA was reverse transcribed with High Capacity cDNA Reverse Transcription Kit (Applied Biosystems, Foster City, California, USA).

### Real-Time Polymerase Chain Reaction (RT-PCR) analysis

cDNA of lung cancer cells was amplified with the Taqman gene expression assays (Applied Biosystems, Thermo Fisher Scientific, Waltham, Massachusetts, USA): *SERPINA1* (ID: Hs00165475_m1), *POLR2A* (ID: Hs00172187_m1), *TLR4* (ID: Hs00152939_m1), *IL6* (ID: Hs00985639_m1), *CLU* (ID: Hs00156548_m1), *TGFB1* (ID: Hs00998133_m1), *CASP3* (ID: Hs00234387_m1), *TNFA* (ID: Hs01113624_m1) using TaqMan Gene Expression Master Mix (Applied Biosystems) and the fluorescence reader StepOnePlus Real-Time PCR System (Applied Biosystems) according to the protocol of the manufacturer. Data were normalized to *POLR2A*, and relative mRNA levels (ΔCt) were calculated.

### Western blots

Cell culture supernatants were harvested and centrifuged to get cell-free supernatants. Cells were lysed in RIPA buffer (20 mM Tris-HCl pH 7.5, 150 mM NaCl, 9.5 mM EDTA, 1% Triton X-100, 0.1% SDS, and 1% sodium deoxycholate) (Sigma-Aldrich, St. Louis, Missouri, USA) supplemented with protease inhibitor cocktail (Sigma-Aldrich). To analyse the phosphorylation status of some proteins, the cells were lysed with M-PER Mammalian Protein Extraction Reagent (Thermo Fisher Scientific, Waltham, Massachusetts, USA) supplemented with 1x halt protease and phosphatase inhibitor cocktail (Thermo Fisher Scientific). Lysate protein concentrations were assessed using the BCA protein assay (Pierce, Thermo Fisher Scientific, Waltham, Massachusetts, USA) and protein contents were measured using a Infinite 200 PRO plate reader (Tecan, Männedorf, Switzerland). Equal amounts of lysate protein or equal volumes of cell-free culture supernatant were separated by 7.5% or 10% SDS-polyacrylamide gels prior to transfer onto a polyvinylidene difluoride (PVDF) membranes (Merck Millipore, Burlington, Massachusetts, USA). Membranes were blocked for 1 h with 5% low fat milk (Carl Roth, Karlsruhe, Germany) followed by overnight incubation at 4 °C with primary antibodies (Table 1). The immune complexes were visualized with appropriate horseradish peroxidase–conjugated antibodies (Dako A/S, Glostrup, Denmark) and Clarity ECL western blotting substrate (Bio-Rad, Hercules, California, USA). For western blot image acquisition we used the ChemiDoc Touch Imaging System (Bio-Rad) All images were taken at the optimal auto exposure and processed using Image Lab version 5.2.1. software (Bio-Rad). For the semi quantification of the target protein band we generated the normalized signal intensity value. For this, the signal intensity of the target protein band in each lane was divided by the β-actin (normalization factor or loading control) for that lane. Afterwards, the normalized signal for each target protein band was divided by the normalized target signal observed in the control sample. These ratios expressed the abundance of the target protein as a fold change relative to the control.

**Table 1:** Antibodies used for western blots.

Primary Antibody	Supplier	Dilution
Polyclonal Rabbit Anti-Human Alpha-1-Antitrypsin	Dako, Glostrup, Denmark	1:800
AKT/MAPK Signaling Pathway Antibody Cocktail (ab151279), rabbit IgG	Abcam, Cambridge, UK	1:250
Anti-LC3β (G-9), mouse monoclonal IgG	Santa Cruz Biotechnology, Heidelberg, Germany	1:500
Anti-SQSTM1 (D-3), mouse monoclonal IgG	Santa Cruz Biotechnology, Heidelberg, Germany	1:500
Anti-phospho-Ribosomal Protein S6 (50.Ser 235/236), mouse monoclonal IgG	Santa Cruz Biotechnology, Heidelberg, Germany	1:500
Anti-Flightless I, mouse monoclonal IgG	Santa Cruz Biotechnology, Heidelberg, Germany	1:500
Anti-hCaspase 3, MAB707, mouse monoclonal IgG	R&D Systems, Biotechne, Minneapolis, Minnesota, USA	1:250
Human TLR4 antibody, AF1478, goat polyclonal IgG	R&D Systems, Biotechne, Minneapolis, Minnesota, USA	1:200
Anti-β-Actin-HRP conjugated	Sigma-Aldrich, St. Louis, Missouri, USA	1:20000

### ELISA

Cell-free supernatants were analysed with human IL-6 DuoSet ELISA set (detection range: 9.38–600 pg/ml; R&D Systems, Minneapolis, Minnesota, USA).

### Cell migration

Cellular migration was determined by the Transwell assay using 8-μm pore size membrane filters in 24-well chemotaxis chambers (Corning Transwell PC inserts, Lowell, MA, USA) as described elsewhere^18^. Shortly, cells were suspended in serum-free medium and added to the upper chamber (1×50^4^ cells, 250 μl). HBSS, STS (50 nM), or AAT (1.5 mg/ml) alone, or in combination was added to the cells. Complete medium supplemented with 10% FCS was added to the lower chamber. Following incubation at 37 °C for 3 h, the cells migrated to the lower side of the insert were fixed in 100% methanol and stained with 0.1% crystal violet. For quantification, stained cells were counted under a microscope (Leica DM750 equipped with Leica ICC50 HD camera, Leica Microsystems, Wetzlar, Germany) with 10x objective from 6 fields.

### Lactate Dehydrogenase (LDH) cytotoxicity assay

To monitor cytotoxicity LDH release into the culture supernatant was analyzed using LDH-Cytotoxicity Detection Kit (Roche, Basel, Switzerland). Samples were measured with Infinite 200 PRO Microplate reader (Tecan, Männedorf, Switzerland). Individual samples were run in duplicates. Experiments were repeated three times.

### Apoptosis assay

Apoptosis was assessed using the PE Annexin V Apoptosis Detection Kit (BD Pharmingen, San Diego, California, USA) according to the manufacturer’s instructions. Briefly, the cells were harvested by trypsination and stained with PE Annexin V and 7-AAD. Cell apoptosis was analyzed in a flow cytometer (Guava easyCyte Flow Cytometer, Luminex, Austin, Texas, United States). Cells that are negative for Annexin V and 7-AAD (bottom left quadrant) were classified as “living”.

### Ki-67 Immunofluorescence staining

Cells were grown on coverslips. At 70% of confluency, the cells were fixed with 4% paraformaldehyde for 30 min at room temperature, permeabilized with 0.1% Triton X-100 (Sigma-Aldrich, St. Louis, Missouri, USA) in PBS for 10 min and blocked for 30 min with blocking solution (0.3% glycine (Carl Roth, Karlsruhe, Germany), 1% bovine serum albumin (BSA, Merck Millipore, Burlington, Massachusetts, USA), 5% goat-serum (Gibco, Thermo Fisher Scientific, Waltham, Massachusetts, USA), 0.1% Tween-20 (Carl Roth, Karlsruhe, Germany) in PBS). The coverslips were incubated for 1 h with primary monoclonal rabbit anti-human Ki-67 antibody (1:500 in blocking solution, R&D Systems, Minneapolis, Minnesota, USA) followed by AlexaFluor-488-labeled goat anti-rabbit IgG (1:1000 in blocking solution, Life Technologies Corporation, Carlsbad, California, USA) for 1 h. Afterwards, slides were mounted using DAPI containing medium (4’−6-diamidino-2-phenylindole, Thermo Fisher Scientific, Waltham, Massachusetts, USA). Images were made with the iRiS Digital Cell Imaging multicolour fluorescence system (Logos Biosystems, Villeneuve d’Ascq, France).

### CyQUANT NF Cell Proliferation Assay

To quantify the cell proliferation of cancer cells the CyQUANT NF Cell Proliferation Assay Kit (Thermo Fisher Scientific, Waltham, Massachusetts, USA) was used according to the supplier’s instruction. Cells were trypsinized, suspended in growth medium and seeded in a density of 500 cells in 100 µl in 96-well microplates (500 cells in 100 µl). After 40 h the medium was aspirated and the cells were incubated with 50 µl dye solution (1x HBSS buffer supplemented with CyQUANT NF dye reagent and dye delivery reagent). After an incubation of 40 minutes at 37 °C the fluorescence intensity was measured in Infinite 200 PRO Microplate reader (Tecan, Männedorf, Switzerland).

### Statistical analyses

Student’s t-test was applied to compare two sample means on one variable. When more than two groups were involved in the comparison, one way ANOVA was used. Data were presented as mean (SD) but if normality test failed, the nonparametric Kruskal-Wallis One Way Analysis was performed and data were presented as median (25th to 75th percentile). A p-value of less than 0.05 was considered significant. All statistics and pairwise multiple comparison were performed using Sigma Plot 14 software package. Data were visualized with GraphPad Prism 5 (GraphPad Software, San Diego, California, USA).

## Supplementary information

Supplementary Figure S1.

Supplementary information .

Supplementary Figure legend.
