# Supplementary figures and images for: Alpha1-antitrypsin protects lung cancer cells from staurosporine-induced apoptosis: the role of bacterial lipopolysaccharide
Source: Sci Rep. 2020 Jun 12;10:9563. doi: 10.1038/s41598-020-66825-w (PMC7293251; doi:10.1038/s41598-020-66825-w)

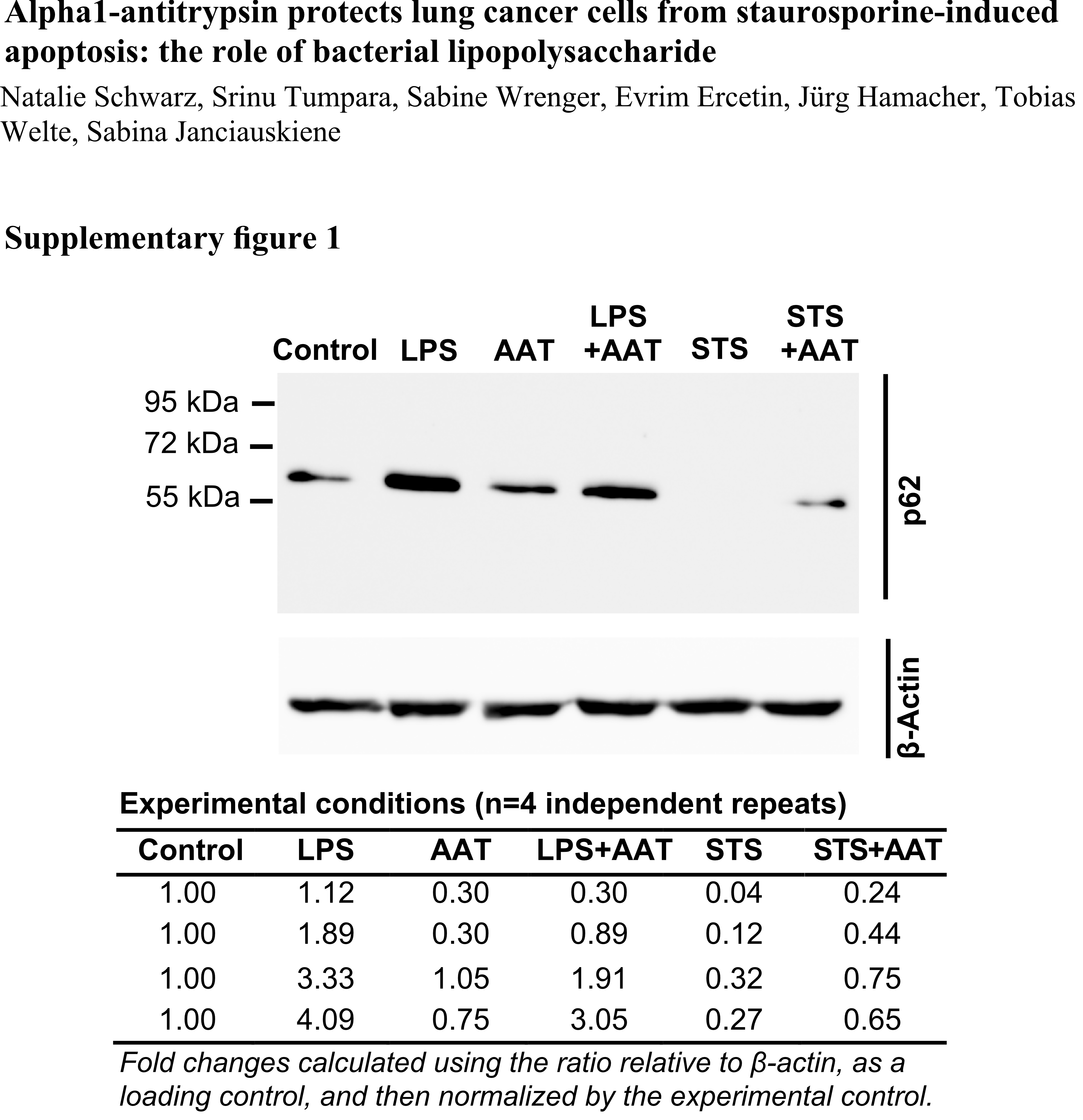

Supplement: Supplementary file 1 — Supplementary Figure S1. [file 41598_2020_66825_MOESM1_ESM.tiff]
