# Supplementary material for: Alpha1-antitrypsin protects lung cancer cells from staurosporine-induced apoptosis: the role of bacterial lipopolysaccharide
Source: Sci Rep. 2020 Jun 12;10:9563. doi: 10.1038/s41598-020-66825-w (PMC7293251; doi:10.1038/s41598-020-66825-w)

Alpha1-antitrypsin protects lung cancer cells from staurosporine-induced apoptosis: the role of bacterial lipopolysaccharide

Natalie Schwarz[1*], Srinu Tumpara[1*], Sabine Wrenger[1], Evrim Ercetin[1], Jürg Hamacher [2,3], Tobias Welte[1], Sabina Janciauskiene[1]

[1]Department of Internal Medicine, Biomedical Research in Endstage and Obstructive Lung Disease Hannover (BREATH), Member of the German Center for Lung Research (DZL), Hannover Medical School, 30625 Hannover, Germany
[2]Pneumology, Clinic for General Internal Medicine, Lindenhofspital Bern, 3012, Bern, Switzerland.
[3]Lungen-und Atmungsstiftung, Bern, 3012, Bern, Switzerland.
* These authors contributed equally

Correspondence:
Prof. Dr. Sabina Janciauskiene
Department of Internal Medicine,
Hannover Medical School
Feodor-Lynen Str.23
30625 Hannover, Germany
Tel: +49-511-532-7297
e-mail: janciauskiene.sabina@mh-hannover.de

Full Western Blots:

Figure 6 A

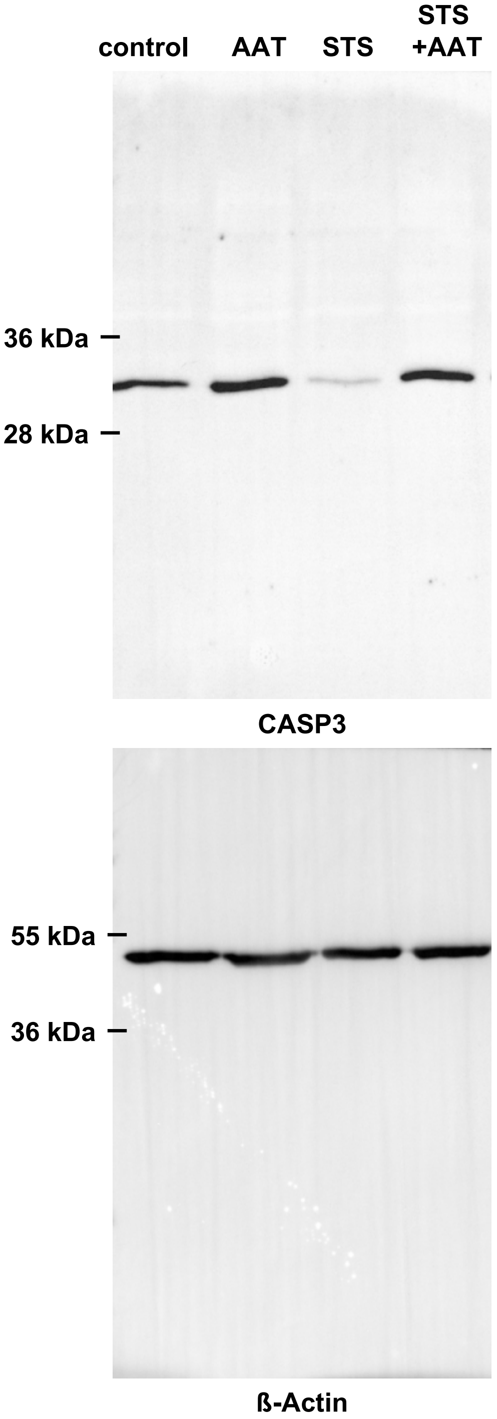
control
AAT
STS
STS +AAT
36 kDa
28 kDa
CASP3
55 kDa
36 kDa
ß-Actin

Figure 7 A

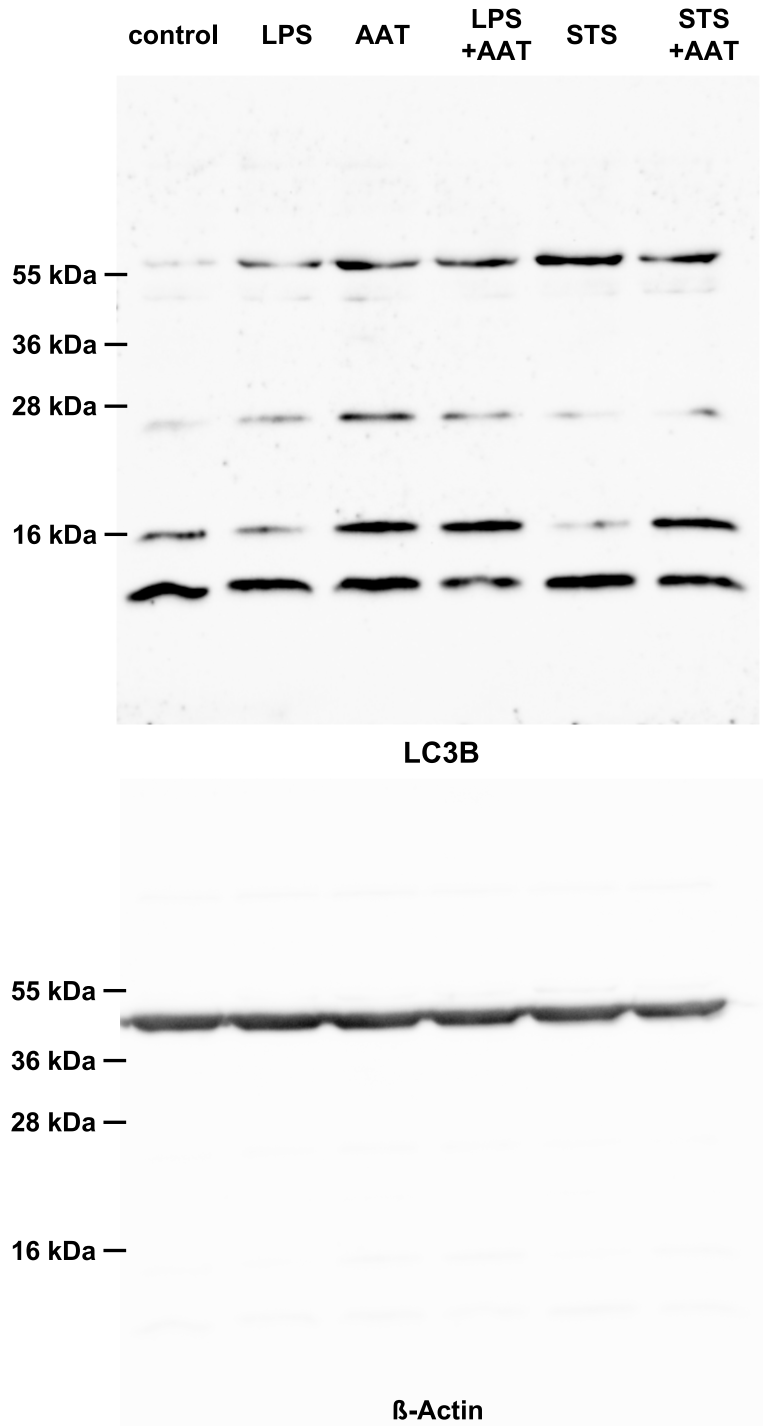

Figure 7 B

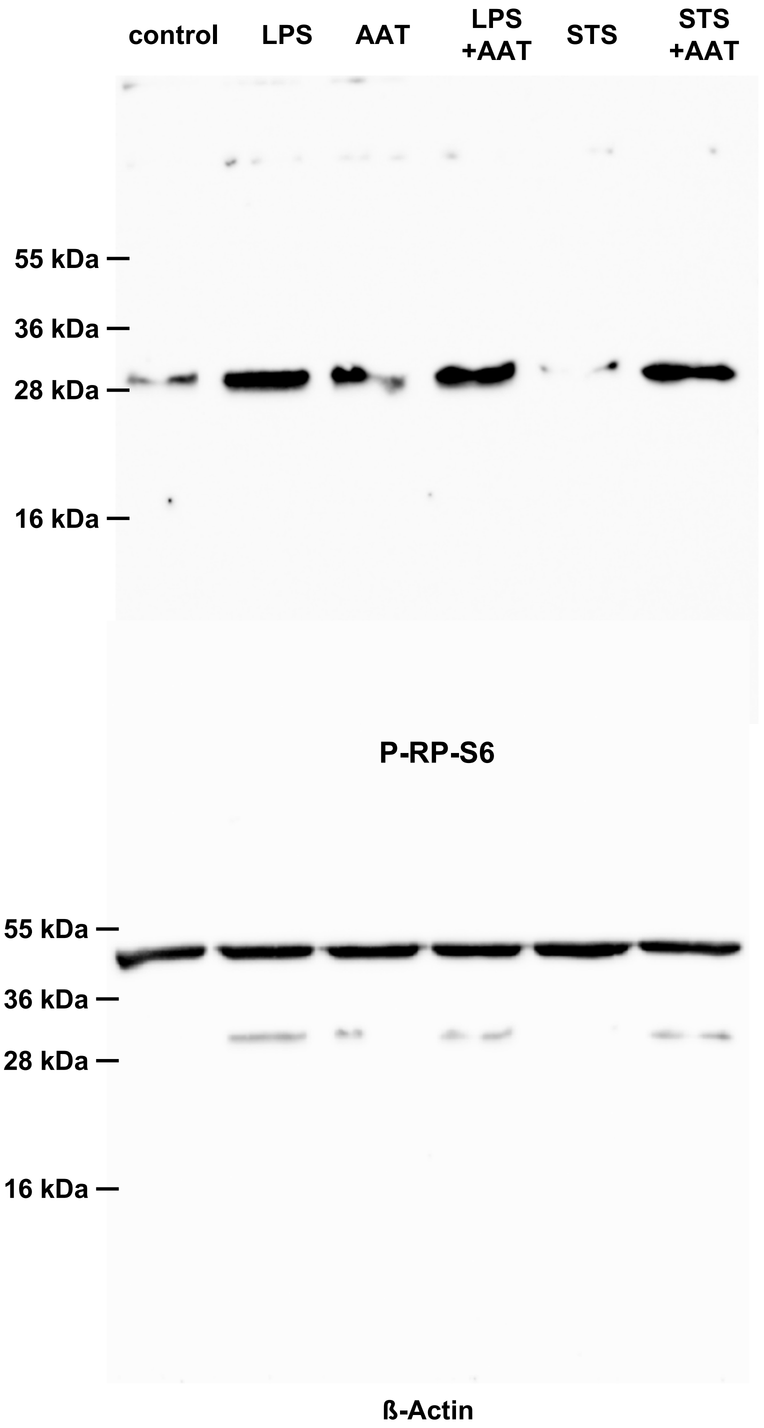

Figure 7 C

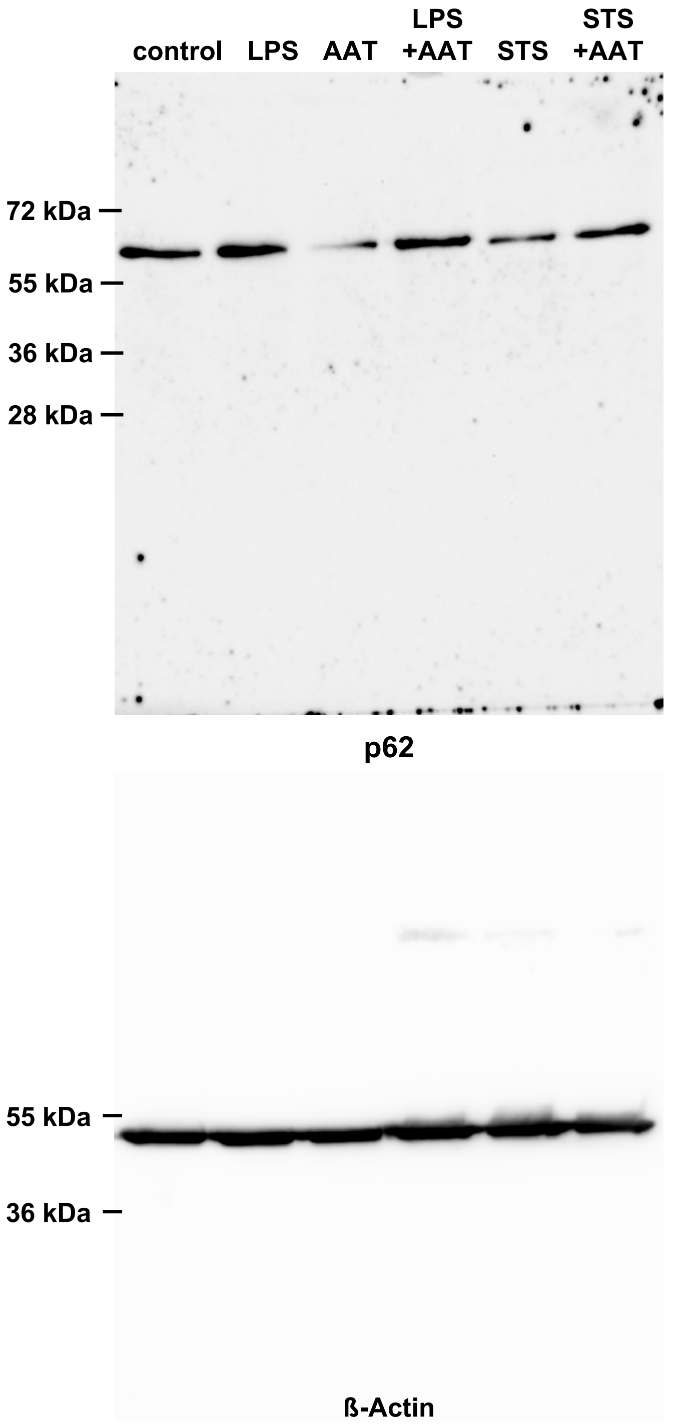

Figure 7 D

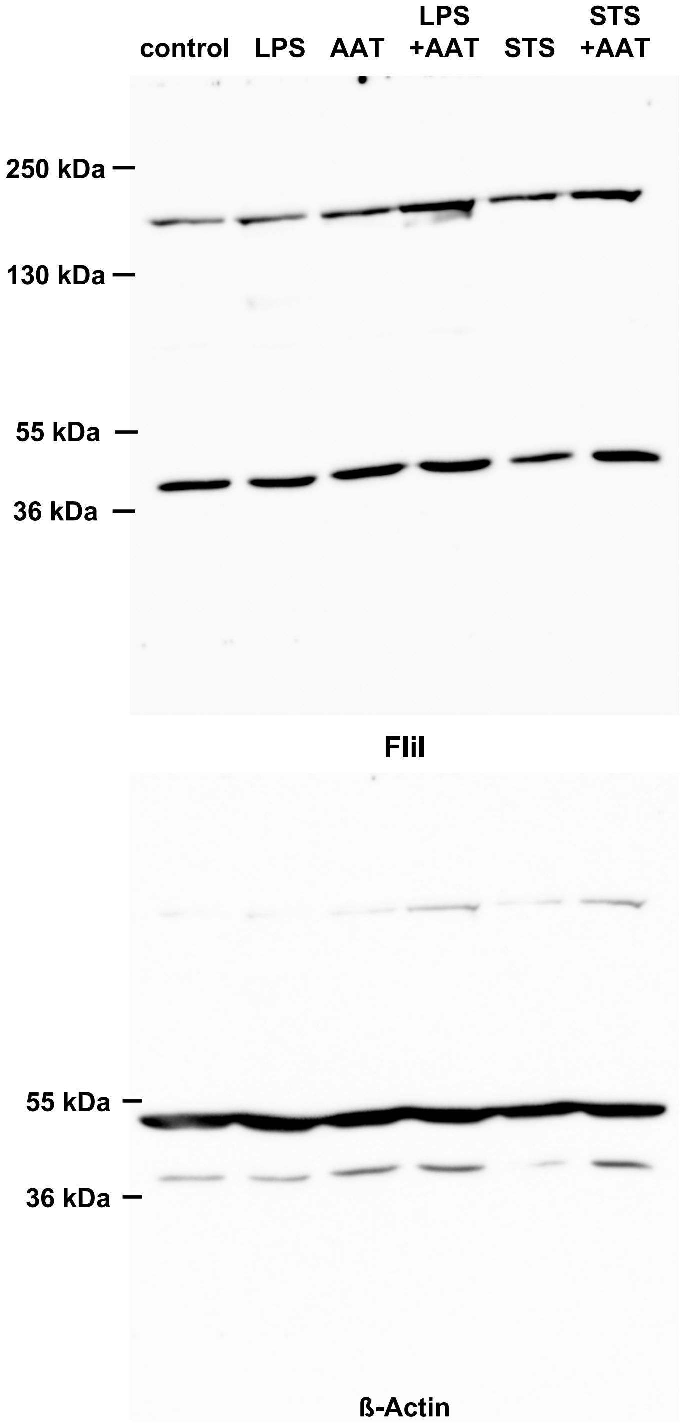

Figure 9 B & E

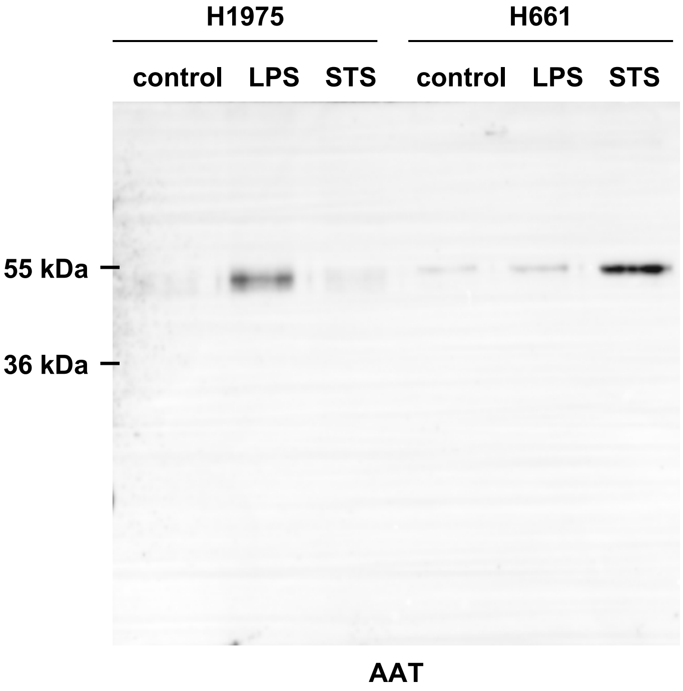

Figure 9 C & F

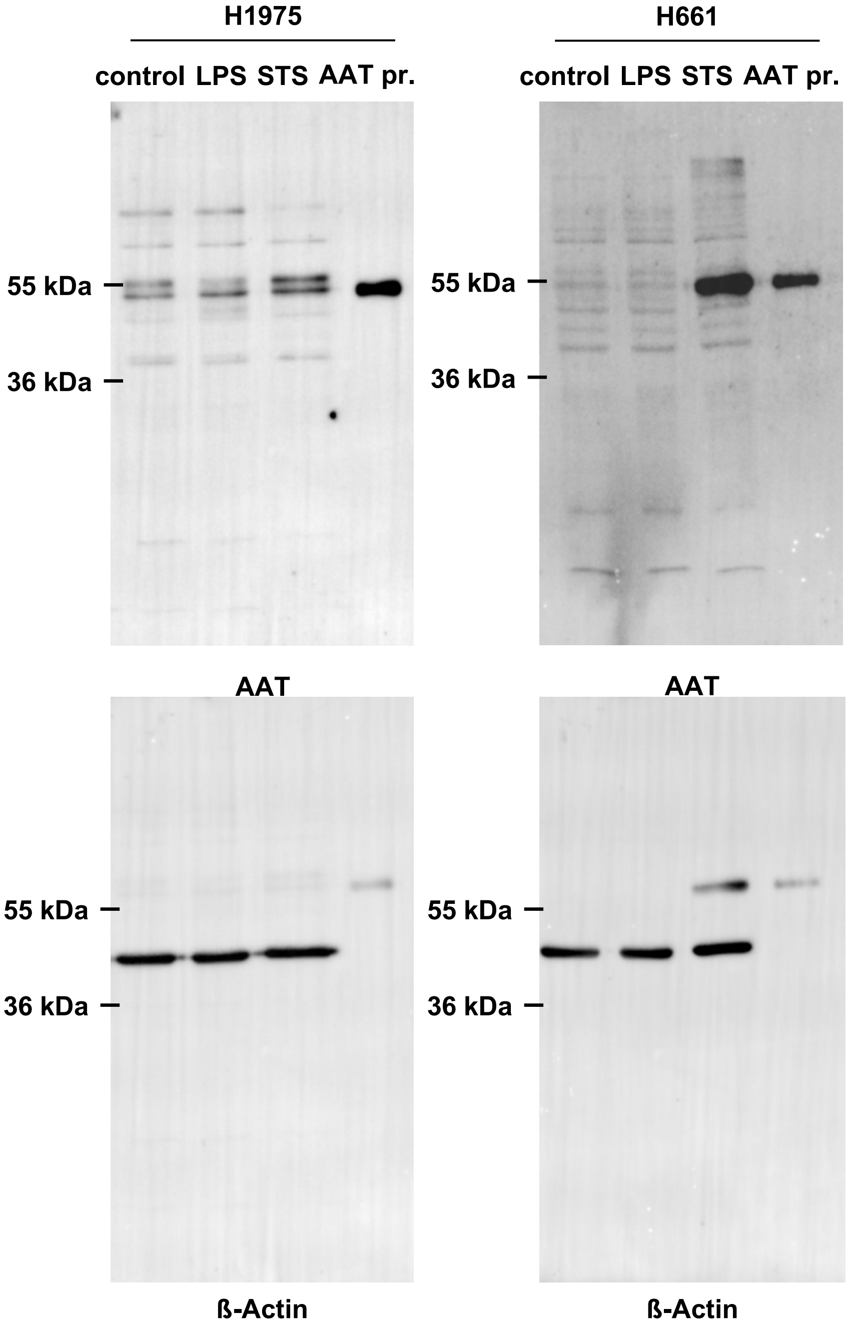

Figure 11 B

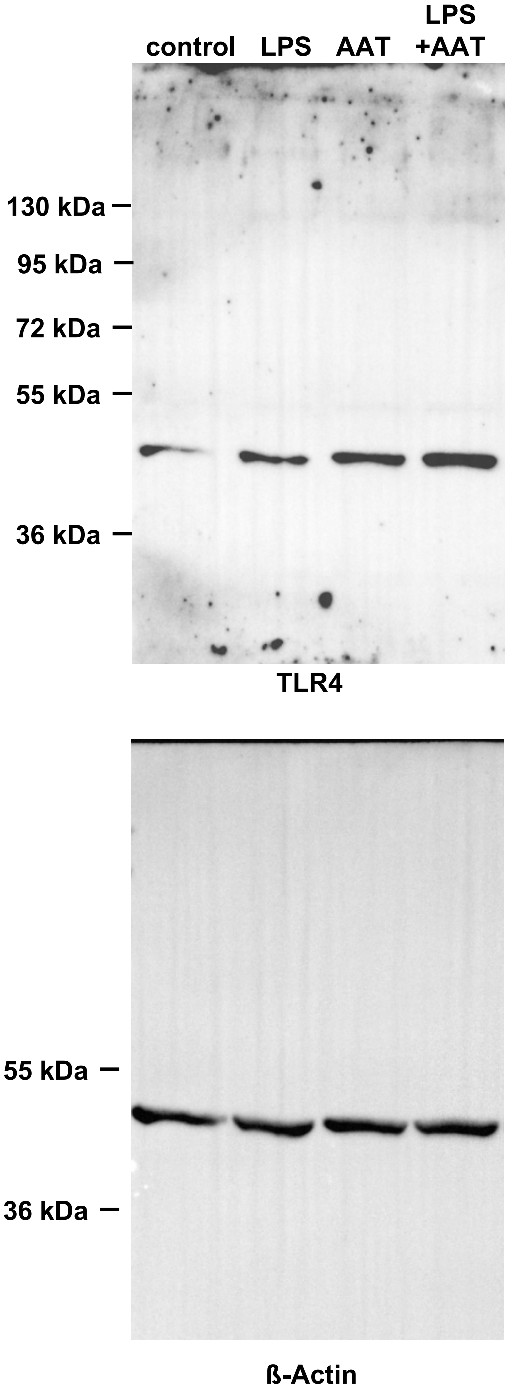
control
LPS
AAT
LPS +AAT
130 kDa
95 kDa
72 kDa
55 kDa
36 kDa
TLR4
55 kDa
36 kDa
ß-Actin

Figure Supp. 1

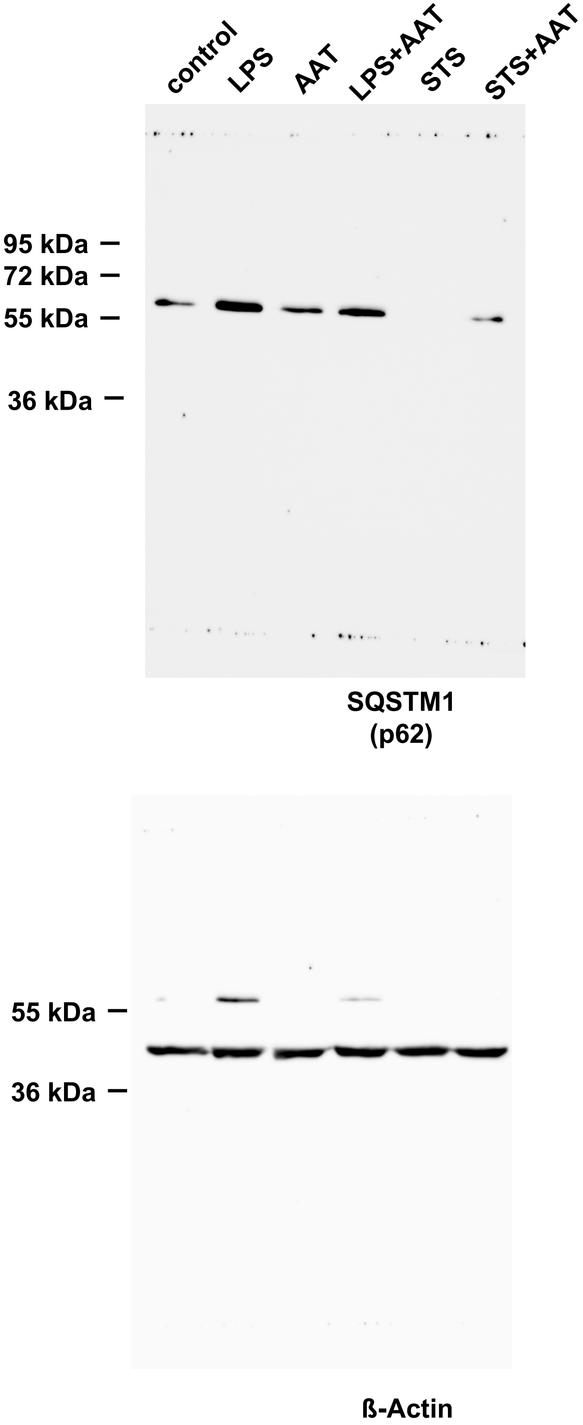

Supplement: Supplementary file 2 — Supplementary information . [file 41598_2020_66825_MOESM2_ESM.pdf]
