# Supplementary material for: Alpha1-antitrypsin protects lung cancer cells from staurosporine-induced apoptosis: the role of bacterial lipopolysaccharide
Source: Sci Rep. 2020 Jun 12;10:9563. doi: 10.1038/s41598-020-66825-w (PMC7293251; doi:10.1038/s41598-020-66825-w)
Supplement: Supplementary file 3 — Supplementary Figure legend. [file 41598_2020_66825_MOESM3_ESM.docx]

**Fig S1**. Western blot analysis of p62 levels in cells treated with AAT, LPS or STS.

H1975 cell were incubated with 50 nM STS, 10 µg/ml LPS, or 1.5 mg/ml AAT, alone or in combination for 18 h. Table shows fold changes from four independent experiments.
